# Serum miRNAs, a potential prognosis marker of loco-regionally advanced nasopharyngeal carcinoma patients treated with CCRT

**DOI:** 10.1186/s12885-020-6689-7

**Published:** 2020-03-04

**Authors:** Zhimin Zhang, Jiangbiao Huang, Ge Wang, Feng Jin, Jijun Zheng, He Xiao, Lin Lei, Jia Luo, Chuan Chen

**Affiliations:** 1 Cancer Center, Institute of Surgery Research, Daping Hospital, Third Affiliated Hospital, Army Medical University (Third Military Medical University), Chongqing, 400042 China; 2 Department of Oncology, General Hospital of Central Theater Command, People’s Liberation Army, Wuhan, 430070 Hubei China; 3 Medical College, Wuhan University of science and technology, Wuhan, 430065 Hubei China

**Keywords:** Nasopharyngeal carcinoma, miRNA, Serum, Concurrent chemoradiotherapy

## Abstract

**Background:**

Serum miRNA was once found as potential disease survival index,thus we investigated the role of miRNA in predicting prognosis in loco-regionally advanced NPC patients treated with CCRT.

**Methods:**

This study included two phases: (i) We enrolled 3 NPC patients with recurrence or distant metastasis (experimental group, EG) and 3 NPC patients in clinical remission (control group, CG),who were treated with CCRT within 5 years. The paired serum was collected before and after treatment and biomarkers were discovered by LNA-TaqMan Human MicroRNA Arrays. (ii) we used the bioinformatic analysis, marker selection and an independent validation by qRT-PCR to analyse the serums of 29 NPC patients with recurrent disease or distant metastasis and 19 NPC patients in clinical remission treated with CCRT. Using the Kaplan-Meier method, log-rank test and Cox regression model to estimate the accuracy of the miRNAs to predict PFS and OS, and identified factors significantly associated with prognosis, respectively.

**Results:**

Using fold change≥2.0 or ≤ 0.5 and *p* ≤ 0.05 as cutoff levels, we identified 1 up-regulated and 6 down-regulated miRNAs, 1 up-regulated and 9 down-regulated miRNAs in EG versus CG before and after CCRT, respectively. After these down-regulated miRNAs were dealed with bioinformatics analysis and normalization, only 5 different miRNAs were significantly reduced, which there were no significant difference in the expression of miRNA-26b, miRNA-29a and miRNA-125b before CCRT, and the expression of miRNA-143 and miRNA-29b after CCRT in the serum samples of 48 NPC patients. Based on this, we calculated a risk score with the expression of miRNA-26b、miRNA-29a、miRNA-125b、miRNA-29b、miRNA-143 and then classified patients as high or low risk group. Cox regression model suggested that combining miRNA-29a and miRNA-125b before CCRT with miRNA-26b after CCRT was independent prognostic factors for PFS (HR = 3.149, 95%CI:1.018–9.115, *p* = 0.034), whereas combining the former two is independent for OS (HR = 5.146, 95%CI:1.674–15.817, *p* = 0.04).

**Conclusions:**

For loco-regionally advanced NPC patients treated with CCRT, especially high-risk patients- serum miRNAs, such as miRNA-29a, miRNA-125b and miRNA-26b etc., play an important role in predicting prognosis factors of PFS and OS, which will contribute to the strategic direction for future research.

## Background

Nasopharyngeal carcinoma (NPC) is endemic in the Far East, particularly in Southern China. NPC is more sensitive to radiotherapy than other head and neck cancers, which results in 5-year overall survival rates from 32 to 52% [1]. However, local recurrences following radiotherapy and high affinity for distant metastasis are still two major causes of treatment failure. The search for non-invasive tools for the diagnosis and management of NPC after radiotherapy has long been a goal of cancer research, which has led research to focus on circulating nucleic acids in plasma and serum.

MicroRNAs (miRNAs) are a class of endogenous small noncoding RNAs, approximately 22 nucleotides in length. They are known to negatively regulate gene expression or destroy the stability of genes via incomplete or complete matching with the 3′UTR of their target genes at the post-transcriptional level [2]. Evidence suggests that miRNA expression profiles can cluster similar tumour types together more accurately than protein-coding mRNA genes profiles. Hence, the most promising application of miRNAs might permit to assess the outcome and modification of response in known and well established anti-tumour therapies (radiation and chemotherapy [3]). Furthermore, a lot of studies have shown that tumour-associated miRNAs are in the serum or plasma of patients suffering from breast, colon, colorectal and nasopharyngeal cancers, etc. [4]. In addition, the meta-analysis showed a possible impact of miRNA expression on NPC patients’ survival outcomes. They even pointed out to 65 miRNAs which have potential to function as prognostic markers in NPC. Further large-scale prospective studies about the clinical significance of the miRNAs may be necessary in order to obtain conclusive results [5]. As a result, using miRNA as a novel noninvasive molecular marker for prognosis prediction of cancer treatment is possible. On the one hand, the current study aimed at finding out if specific circulating miRNAs can be detected in serum,and meanwhile if specific miRNA expression level differ in NPC patients (treated by concurrent chemoradiotherapy) with recurrence or metastasis and without. To our knowledge, this study is the first to evaluate the feasibility of using serum miRNAs as a noninvasive prognostic prediction test in loco-regionally NPC patients treated with CCRT.

## Methods

### Study design and patient samples

Written informed consent, was obtained from all patients. It included the permission to use blood for research purposes. The study was approved by institutional review boards and the hospital Clinical Research Ethics Committee. All patients were sporadic cases on the basis of family history of NPC. All patients first underwent neoadjuvant chemotherapy as described previously [6]. The treatment consisted of neoadjuvant chemotherapy and concurrent radiotherapy. Chemotherapy was made of 2 cycles of 5- fluoroucilat(5-FU) 700 mg/m^2^/a day, performed on day 1 and day 4 (intraveneous injection). In addition, on day 1 after fluoroucilat, nedaplatin was infused (100 mg/m2 over 2 h). Nedaplatin treatment was repeated every 3 weeks, on days 1, 22 and 43, respectively, which was given 60 min before concurrent intensity modulated radiotherapy. Radiotherapy consisted of external-beam radiotherapy to the nasopharynx (70–80 Gy), the lymph node–positive area (60–70 Gy), and the lymph node–negative area (50–60 Gy). Tumours were staged according to the 1997 American Joint Committee on Cancer (AJCC) Staging system.

#### Inclusion criteria

Patients were included when they were between 40 and 70; radiotherapy was indicated for them; they had no endocrinologic or metabolic disorders and no uncontrolled hypertension or infections. They needed to have a normal liver, heart and kidney function.

#### Exclusion criteria

Patients could not be included when they were intolerant to radiotherapy;they had not finished a prescribed treatment;they were unable to accept treatment.

### This study was divided into two phases

- **Phase I (Marker discovery):** In this phase, 6 patients with loco-regionally advanced NPC underwent intensity-modulated radiotherapy (IMRT) concurrently performed with induction chemotherapy based on nedaplatin (NDP) and 5-FU. Serum samples were collected within the week before radiotherapy initiation and three months after radiotherapy completion. Among these 6 patients, 3 experienced recurrence or distant metastasis in the 5 years after chemoradiotherapy, who belonged to the experimental group (EG);while the other 3 NPC patients experienced complete clinical remission in the 5 years after chemoradiotherapy,who belonged to the control group (CG). Differences in miRNA profiles between EG and CG groups collected before and after chemoradiotherapy were assessed in serum samples. Two miRNA expression patterns were established by comparing miRNA profiles in these two groups using miRCURY LNA™ miRNA Arrays. The most frequently down-regulated miRNAs in CG group compared with EG group in both time points were identified by bioinformatics and used for further analysis in phase II.
- **Phase II (Marker selection and validation):** The down-regulated miRNAs identified above were considered as possible candidates. Two batches of serum samples were collected from an independent cohort of 48 NPC patients within 7 days before the start of radiotherapy and three months after radiotherapy completion.29 NPC patients with recurrence or distant metastasis and 19 NPC patients in clinical remission within 5 years after CCRT composed the cohort. Putative miRNA markers identified in phase I were verified in these independent sets of serum samples via real-time quantitative RT-PCR.

### miRNA array analysis

Trizol LS reagent (Invitrogen, Paisley, UK) was used to extract RNA. miRNAs were generated from the total RNA groups mentioned above. miRCURY locked nucleic acid (LNA) microarray platform (Exiqon, Denmark) was used [7]. Total RNA was labeled Hy3™ or Hy5™ fluorophores, using miRCURY™ Array Power Labeling kit (Exiqon, Denmark). Then, the reaction was spinned and left at 4 °C. The two samples from the Hy3™ and Hy5™ labeling reactions were combined in ice. The samples were hybridized in an hybridization station using miRCURY™ LNA miRNA Array (v.11.0) containing Tm–normalized probes for 847 human miRNAs. Microarrays with labeled samples were hybridized at 56 °C overnight using a heat-shrunk hybridization bag and washed with miRCURY Array Wash buffer kit (Exiqon, Denmark). After hybridization, the chip slides were immediately washed, dried and scanned. Each miRNA spot was replicated four times on the same slide and two microarray chips were used for each group. Scanning was performed with the Axon GenePix 4000B microarray scanner. GenePix pro V6.0 was used to read the raw intensity of the image. Signal intensities for each spot were scanned to produce the best within-slide normalization and minimize the intensity-dependent differences between the dyes. Then, they were calculated by subtracting local background (based on the median intensity of the area surrounding each spot) from total intensities using locally weighted scatter plot smoothing (Lowess, Locally Weighted Scatter plot Smoothing) Normalization (MIDAS, TIGR Microarray Data Analysis System). After normalization, the average values of each miRNA spot were used for statistics. The ratio between the green and red signals was calculated. Fold change > 2.00 or fold change < 0.5 (adjusted *p*-value< 0.50) were used to screen both up and down regulated miRNAs. Hierarchical clustering to differentiate expressed miRNAs was generated using standard correlation to measure their similarity.

### Real-time quantitative PCR

Real-time PCR was done using GeneAmp Fast PCR Master Mix (Applied Biosystems) and ABI 7900HT real-time PCR machine. Table 1 summarizes the Oligonucleotides used in this study. To quantify the miRNAs expression in serum, U6 or miRNA-16 was respectively adopted as internal control. Appropriate internal normalization control was required to normalize sample-to-sample variations and relative quantification was applied in this qPCR. As no consensus on the use of internal normalization control in serum was defined for miRNA qPCR, we used expressions of miRNA-16 as the internal normalization control for miRNA quantification in serum. Although miRNA-16 turns less abundant than other miRNAs in the serum, miRNA-16 was selected as the normalization control as it proved higher stability and less variability. The threshold cycle (Ct) is defined as the fractional cycle number at which the fluorescence passes the fixed threshold. Each sample was run in triplicates for analysis. The relative amount of each miRNA was calculated using the eq. 2^-ΔCt^, in which ΔCT = (CT^miRNA^-CT^U6^) or ΔCT = (CT^miRNA^-CT^miRNA-16^).

**Table 1:** Oligonucleotides used in this study

Primer set name	Reverse transcripatase reation primer	Real-time quantitative PCR primer	Tm (°C)	Length (bp)
U6	5’CGCTTCACGAATTTGCGTGTCAT3’	Forward:5’GCTTCGGCAGCACATATACTAAAAT3′ Reverse:5’CGCTTCACGAATTTGCGTGTCAT3’	60	89
miR-16	5’GGCGTAGGCAGTGCAGGGTCCGAGGTCTGCCTACGCCCGCCAATA3’	16-F:TCGGCTAGCAGCACGT; 16-R:TGATTGCAGGGTCCGAG	60	60
miR-29a	5’GCGTGGTCGGTAACTCGGACCCTTCTACCGACCACGCTAACCGA 3’	29a-F:GAACCCCTTAGCACCATCT; 29a/b-R:AGCGTAACTCGGACCCTT	60	66
miR-29b	5’GCGTGGTCGGTAACTCGGACCCTTCTACCGACCACGCAACACTG3’	29b-F:GAACCCCTTAGCACCATTT; 29a/b-R:AGCGTAACTCGGACCCTT	60	66
miR-26b	5’GCCGTGACCGTCAGTGGAGGCAAGCCAGACGGTCACGGCACCTAT 3’	26b-F:ACGACGGTTTCAAGTAATTCA; 26b-R: TCTCGTCAGTGGAGGCAA	60	65
miR-125b	5’GCCGTACCGTCAGTGGAGGCAAGCCAGACGGTACGGCTCACAAGT 3’	125b-F:CTGGACTCCCTGAGACCCT; 125b-R:ATCCGTCAGTGGAGGCA	60	66
miR-143	5’GGCGTCAGCCAGAGTGGAGGCAAGCCACTGGCTGACGCCGAGCTAC 3’	143-F:GCCAGTCCTGAGATGAAGCAC; 143-R:TGAAGAGTGGAGGCAAGC	60	62

### Analyzing miRNA targets and correlative article

The targeted mRNAs that have the potential binding sites for these miRNA to express in EG versus CG were searched in public databases endowed with prediction algorithms, such as TargetScan (http://targetscan.org), PicTar (http://pictar.mdc-berlin.de) and miRBase Target (http://www.mirbase.org). The target genes we have chosen were significantly associated with different pathways. We also performed a PubMed search with various down-regulated miRNAs in our results from miRNA array analysis. Relevant publications dealing with miRNAs and their possible molecular mechanisms were obtained. Further, relevant articles were found by screening the references of these papers. In case of non-availability of the whole article, the abstract was taken into consideration despite the limited data provided. This procedure was conducted twice until the beginning of May 2019 to avoid any missed contribution.

### Development of risk score

To analyse the data of miRNAs selection, we performed a multivariate Cox regression analysis using a backward stepwise approach to test if the signature was an independent prognostic factor of PFS and OS. The expression level of miRNA-26b, miRNA-29a, miRNA-125b miRNA-29b and miRNA-143 in NPC patients serum before and after treatment were used as candidate variates. PFS was used as dependent variable, *p* < 0.15 was selected as variable standard, and *p* > 0.2 was removed out of variable standard. We developed a formula to calculate every patient’s PFS and OS risk scores from the expression values of the five miRNAs, weighted by regression coefficient.

For PFS, Risk score = (0·792 × expression value of pre-therapy miRNA-29a) –(0·779× expression value of pre-therapy miRNA-125b) + (0·242 × expression value of post-therapy miRNA-26b). For OS: Risk score = (0·431× expression value of pre-therapy miRNA-29a) –(0·538× expression value of pre-therapy miRNA-125b) .

### Statistical analysis

Data about miRNA expression was analyzed using BRB-Array Tools version 3.5.0 software (Richard Simon & BRB-ArrayTools Development Team), TIGR Multi-experiment viewer version 4.0 software (The Institute for Genomic Research, and Array Assist software, Stratagene), R software version 2.15.2 (The R Foundation for Statistical Computing c/o Institute for Statistics and Mathematics) and GraphPad software 5.0 (GraphPad Software Inc., San Diego, CA, USA). Hierarchical clustering (Manhattan distance and average linkage) and principal component analysis (PCA) Student’s *t*-test based on multivariate permutation (with random variance model) were performed to identify miRNAs with significant differential expression between groups. In the validation phases, the expression levels of individual miRNAs in the different groups were compared using paired or unpaired *t*-test or ANOVA analysis for continuous variables. The Spearman rank order correlation test was used to examine correlation relationships between the levels of the miRNA markers. Progression-free survival (PFS) was calculated from the date of the start of a therapy to the documentation of PD according to the RECIST criteria. Overall survival (OS) was defined as the interval between the date when therapy started and the date of the last follow-up or death from any cause. The survival rate was calculated via using the Kaplan–Meier method. Univariable and multivariable Cox proportional hazards models including age, gender, tumour stage (according to the AJCC TNM classification), pathological type, expression level of ki-67 and miRNA were used to identify factors significantly associated with prognosis. Results were reported including hazard ratios (HRs) and 95% confidence intervals (CIs). The data were regarded as significantly different at *P* < 0.05.All statistical calculations were performed by the SPSS software (version 18.0,SPSS Inc., Chicago, IL, USA).

## Results

### Patient characteristics

Table 2 summarizes the baseline characteristics of the studied sample. A total of 48 participants including 29 NPC patients with a recurrence or distant metastasis and 19 cases in clinical remission patients, 5 years after completion of treatment with CCRT were enrolled. No significant differences in each of clinicopathological characteristics were evidenced between NPC patients.

**Table 2:** Patient characteristics for serum microRNA analysis

Parameter		Clinical remission patients		Recurrence or distant metastasis patients			
		N	%	N	%	χ^2^	*P*
Gender	male	15	78.9%	24	82.8%	0.000*	1
	female	4	21.1%	5	17.2%		
Age (years)	≤60	10	52.6%	14	48.3%	0.087	0.768
	> 60	9	47.4%	15	51.7%		
T stage	T1-T2	12	63.2%	12	41.4%	2.178	0.14
	T3-T4	7	36.8%	17	58.6%		
N stage	N0-N1	12	63.2%	18	62.1%	0.006	0.939
	N2-N3	7	36.8%	11	37.9%		
Tumor stage	II-III	9	47.4%	8	27.6%	1.964	0.161
	IV	10	52.6%	21	72.4%		
Pathological type	differentiated	11	57.9%	14	48.3%	1.478	0.153
	undifferentiated	8	42.1%	15	51.7%		
Ki-67	+	13	68.4%	13	44.8%	2.547	0.144
	++	6	31.6%	16	55.2%		

### Identification of therapy-associated miRNAs in serum of NPC patients

**Fig. 1:**
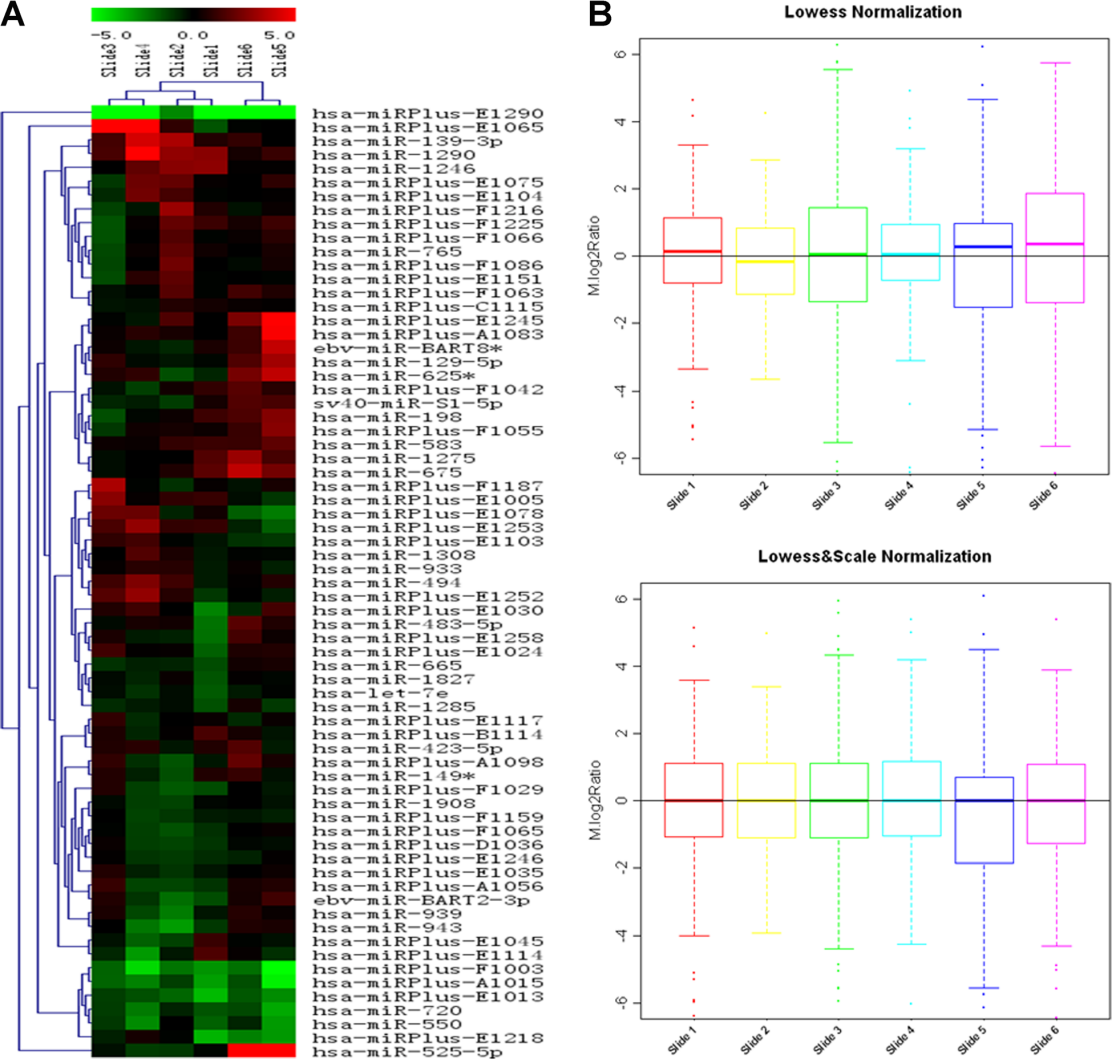
Two-way hierarchical clustering of miRNAs and samples. **a** Data from each miRNA were median centered. Samples are in *columns* and miRNAs in *rows*. The color scale shown at the top illustrates the relative expression level of miRNA in the certain slide: red color represents a higher expression level than control sample; green color represents a lower expression level than the control sample.The actual log2(Hy5/Hy3) ratios for the miRNAs are shown in the expression matrix sheet in slide1~slide6 Data File. **b** Box plot for scale normalization. The box plot shows the miRNA expression profiling before, after and between slide Scale normalization.(top:only within lowest normalization; down:both within and between slide normalization). The main purpose of scale normalization is to control between slide variability. Y-axis represents the log2Ratio M = log2(Hy5/Hy3)

When EG was compared to CG, the microarray-based experiments identified that before radiotherapy, only hsa-miRPlus-E1253 was overexpressed whereas 6 miRNAs were down-regulated. After CCRT, only hsa-miRPlus-E1072 was up-regulated whereas 9 miRNAs were down-regulated when EG was compared to CG (Fig. 1 and Table 3). The *P* values for all these miRNAs were less than 0.05 when EG was compared with CG (Table 3). Based on these differentially expressed miRNAs, a tree with clear distinction between different groups was generated by cluster analysis.

**Table 3:** Up-and Down-regulated miRNAs in serum of NPC patients from EG to CG before and after CCRT

	Name	FoldChange (average)	t-test ***p***-value	Name	FoldChange (average)	t-test ***p***-value
	Before concurrent chemoradiotherapy			After concurrent chemoradiotherapy		
**Down-regulated miRNAs**	miR-29a	0.052	0.0021	miR-29b	0.041	0.009
	miR-26b	0.052	0.0275	miR-143	0.013	0.001
	miR-22	0.068	0.0038	miR-26b	0.013	0.001
	miR-125b	0.089	0.004	miR-22	0.05	0.0003
	miR-720	0.458	0.0256	miR-30c	0.074	0.030
	miR-665	0.489	0.0223	let-7c	0.139	8E-05
				miR-491-3p	0.179	0.049
				miR-720	0.221	0.011
				miR-550	0.294	0.046
**Up-regulated miRNAs**	hsa-miRPlus-E1253^*^	2.297	0.0303	hsa-miRPlus-E1072^*^	2.940	0.037

^*^Unknown function in current miRNA database

### Validation of microarray results using real-time PCR

**Fig. 2:**
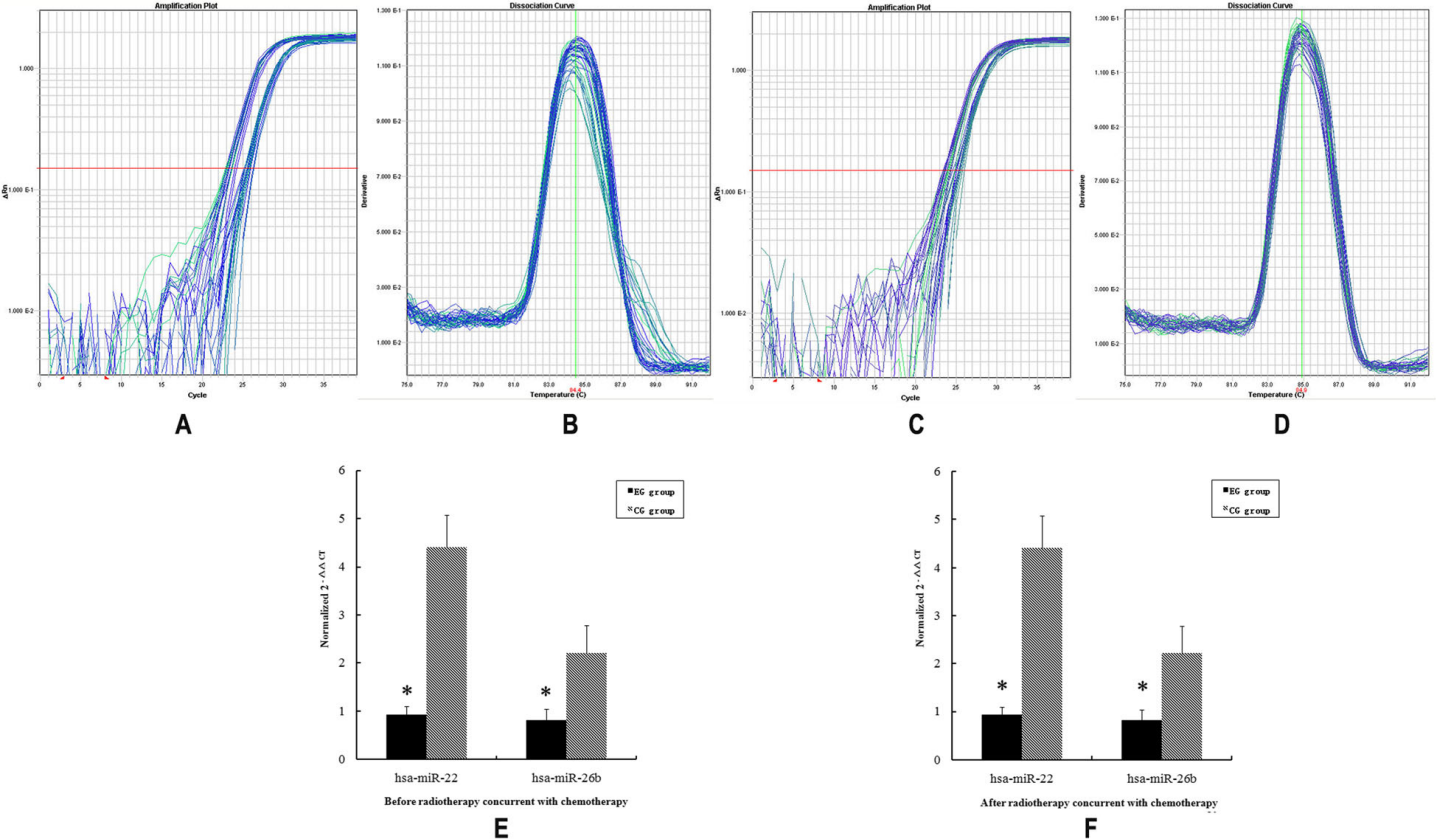
Confirmed miRNA expression by quantitative real-time PCR. **a** Amplification plots of *miRNA-22*; **b** Disassociation curves of *miRNA-22*; **c** Amplification plots of *miRNA-26b*; **d** Disassociation curves of *miRNA-26b*; E, Before NPC patients treated with CCRT, *miRNA-22 and miRNA-26b* were down-regulated in serum from EG versus CG; **f** After treated with CCRT, *miRNA-22 and miRNA-26b* were down-regulated in serum from EG versus CG. The experiment was conducted in triplicate and the relative expression level of each miRNA was normalized to RNU6B. The result is consistent with the microarrays. * *P* < 0.05 versus CG group

To validate the microarray results, quantitative RT–PCR analysis of serum was performed for miRNA-22, miRNA-26b. The RT-PCR analysis is consistent with microarray data (Fig. 2). The expression of miRNA-22 and miRNA-26b was down-regulated in EG versus CG both before and after CCRT serum samples.

### Bioinformatics study of down-regulated miRNAs in microarray results

We used Pubmed search to summarize the most significant and updated findings from original researches about down-regulated miRNAs involvement in our microarray results. We focused on the potential of NPC therapy-related miRNAs as biomarkers for prognosis (Additional file 1). Moreover, to assess the global impact of these down-regulated miRNAs, we conducted a high stringency target prediction to identify potential target genes and then examined the molecular mechanisms, which were specifically enriched with these miRNAs. Results from the pathway enrichment analysis suggested that miRNA-26b, miRNA-29a, miRNA-29b, miRNA-143 and miRNA-125b selected and targeted signaling cascades involved in cancer cell apoptosis, invasion, metastatic and proliferation. These miRNAs need to be further validated in the next stage. Furthermore, the experimental data of six miRNAs (miRNA-22, miRNA-30c, miRNA-550, miRNA-665, miR491-3p and let-7c) was not present in the relevant article, this is the reason why these miRNAs were excluded from further analysis.

### miRNAs selection and validation as a marker in a small set of serum samples

**Fig. 3:**
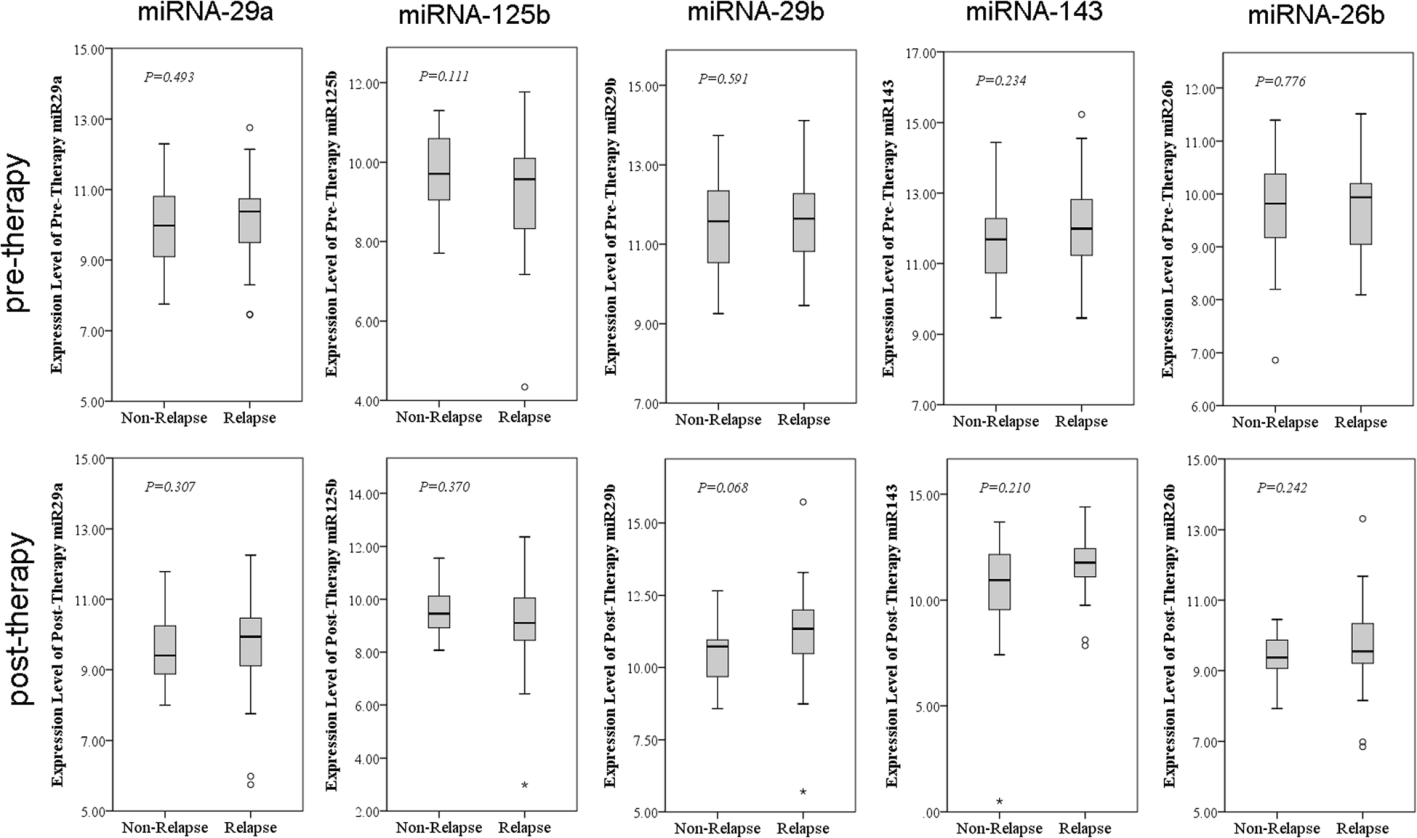
Different serum levels of miRNAs in NPC patients with or without recurrence or distant metastasis. Data showed that the difference in the expression level of miRNA-26b, miRNA-29a and miRNA-125b was not significant when NPC with recurrence or distant metastasis and without before CCRT were compared. Whereas the difference in the expression level of miRNA-143 and miRNA-29b was not significant between the two groups after CCRT. The experiment was conducted in triplicate and the relative expression level in each miRNA were normalized to miRNA-16. * *P* < 0.05 versus NPC cases of clinical remission patients

In this phase, qRT-PCR assays were developed to quantify miRNAs in part of serum samples to validate the putative markers. Using miRNA-16 as normalization control, expression levels of miRNA-26b, miRNA-29a, miRNA-125b miRNA-29b and miRNA-143 were validated by qPCR on the 48 serum samples. As the expression level of miRNA-26b, miRNA-29a and miRNA-125b is concerned, no difference was evidenced between NPC patients before CCRT (*p* > 0.05), which had no significant difference in recurrence or distant metastasis, either (p > 0.05). As to the expression of miRNA-143 and miRNA-29b, no difference was evidenced after CCRT even in case of recurrence or distant metastasis (*p* > 0.05) (Fig. 3).

### A model for predicting survival in loco-regionally advanced NPC patients treated with CCRT

**Fig. 4:**
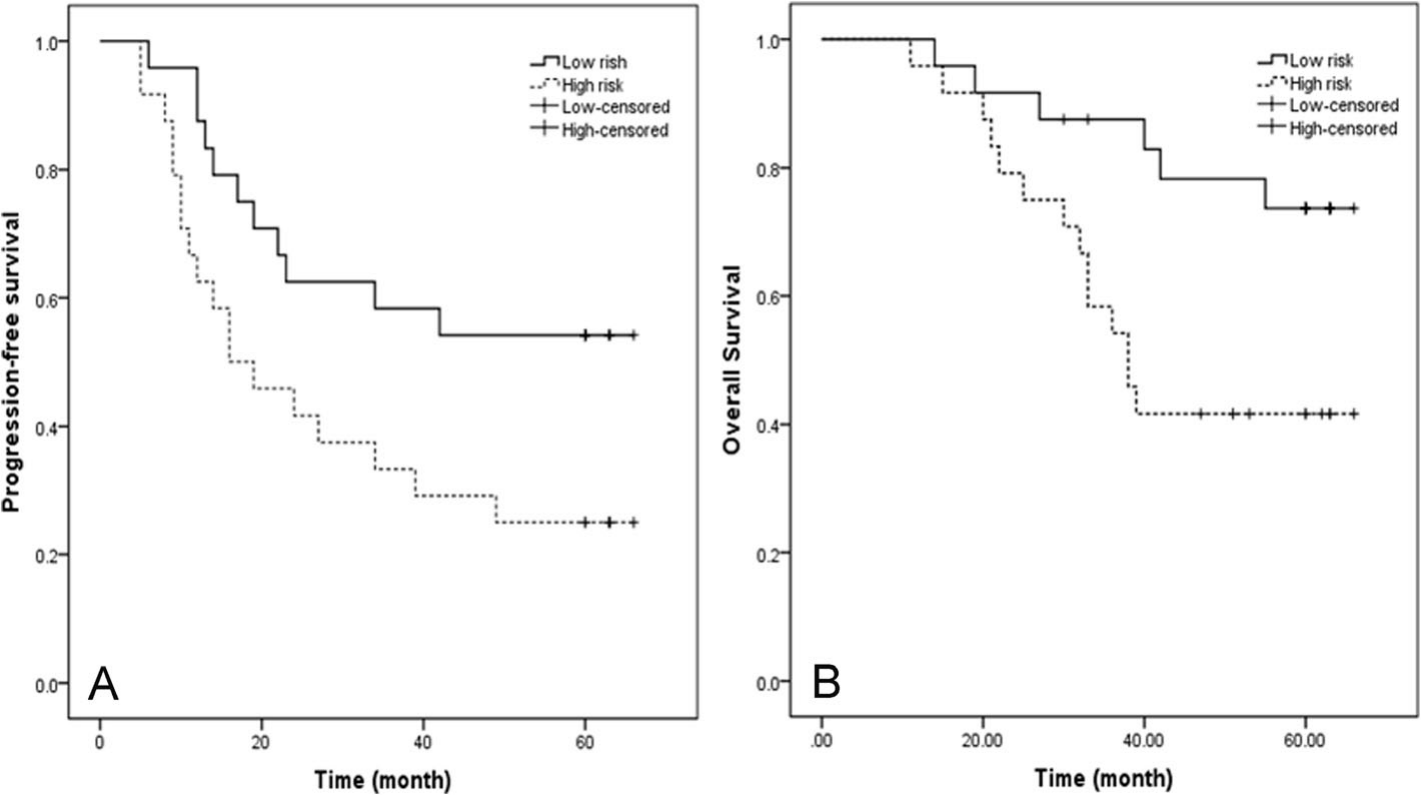
The PFS (**a**) and OS (**b**) according to expression of miRNAs signature in loco-regionally advanced NPC patients with CCRT

Patients in the training set were divided into high-risk or low-risk groups with the median risk score (2.8030) as the cutoff. Compared with patients with low-risk scores, patients with high-risk scores in all the training set had shorter PFS (hazard ratio [HR] 2.263, 95% CI 1.066–4.805; *p* = 0.027) (Fig. 4a). After revising other clinical baseline characteristics, including age, sex, T stage, N stage, tumour stage and expression of Ki-67, the serum miRNA-29a and miRNA-125b before treatment and miRNA-26b after treatment in combination were independent prognostic factors for PFS (high-risk groups vs low-risk groups, hazard ratio [HR] 3.149, 95% CI 1.088–9.115, *p* = 0.034) (Table 4).

**Table 4:** Multivariable analysis of parameters for PFS and OS in loco-regionally advanced NPC patients with CCRT

Parameter	PFS				OS			
	95.0% CI		HR	P	95.0% CI		HR	P
	Lower	Upper			Lower	Upper		
Age	.214	1.717	.607	.347	.182	1.376	.500	.180
Sex	.634	8.513	2.323	.204	.690	10.271	2.662	.155
T stage	.279	3.778	1.027	.968	.214	4.381	.968	.967
N stage	.348	2.391	.912	.851	.317	3.572	1.064	.920
Tumor Stage	.237	5.662	1.158	.857	.141	6.124	.928	.938
Level of Ki67	.746	3.490	1.613	.225	.538	3.687	1.408	.486
Score	1.088	9.115	3.149	.034	1.674	15.817	5.146	.004

With regard to OS, patients were divided into high-risk or low-risk groups with the median-risk score (− 0.7811) as the cutoff. Compared with patients with low-risk scores, patients with high-risk scores in all the training set had shorter PFS (hazard ratio [HR]3.067, 95% CI 1.170–8.044; *p* = 0.016) (Fig. 4b). After revising the same clinical baseline characteristics, serum miRNA-29a and miRNA-125b before treatment in combination were independent prognostic factors for overall survival (high-risk groups vs low-risk groups, hazard ratio [HR] 5.146, 95% CI 1.674–15.817, *p* = 0.004). (Table 4)

## Discussion

Over the past few years, researchers have discovered the particularly important role played for miRNAs in tumourigenesis. Commonly accepted prognostic factors are tumour size, histological grade, lymph node status, and age. So far, useful markers for resistance and/or sensitivity of CCRT have not been identified. Some markers have shown promising results in a limited number of studies, e.g. Ki67 [8], p53 [9], multi-drug resistance-associated protein [10] and circulating tumour cells [11]. Recent studies have revealed that a large number of miRNAs are deregulated in a great variety of tumours [4]. Circulating miRNAs from patients’ serum are definitely promising biomarkers because they can remain stable for a long time. Moreover, they also indicate the nature of the tumor including plasma cells that are either absent or patchy in bone marrow [12].

In the current study, pre- and post-CCRT plasma samples were used to quantify circulating miRNAs in serial serum samples from NPC patients. Furthermore we identified a 9-miRNA signature in NPC patients with or without recurrence or distant metastasis. Whether in clinical remission or having a recurrence or distant metastasis, NPC patients showed an intermediate expression level. Moreover, the expression of five of these miRNAs–miRNA-26b, miRNA-29a, miRNA-125b, miRNA-29b and miRNA-143– down-regulated at the time of treatment– were linked to PFS or OS after CCRT. Importantly, the combination of miRNA-29a and miRNA-125b before treatment and miRNA-26b after treatment retained its prognostic value in the multivariate analysis. The origin and mechanism of the 5-miRNAs are not clear. However, since all these miRNAs are down-regulated in serum, we can speculate that their origin is not in the blood cell. In fact, a recent study has revealed that the serum miRNA showed no correlation with intracellular levels in malignant bone marrow plasma cells in paired samples [13]. Furthermore, the 5-miRNAs have previously been related to tumour suppressor gene, tumour metastasis gene, p53 and NF-KB signalling pathway and so on [14–18]. This led us to speculate that their down-regulation in NPC patients treated with CCRT could be due to the correlation of recurrence or distant metastasis associated with the tumour. Several previous studies have demonstrated that any specific miRNA could play a multi-faceted role (as a tumour suppressor or an oncogene), depending on the tissue or the tumour. This is likely to be due to each specific miRNA targeting multiple mRNAs, and has a different function in an individual cellular context.

Three miRNAs in this study (miRNA-29a, miRNA-125b and miRNA-26b) which are different, have critical functions in various cellular biological activities such as proliferation, apoptosis, invasiveness, metastasis, differentiation, and drug-resistance and so on (Table 3). For example, miRNA-29a has previously been found as a putative tumour-suppressive miRNA which was related to cell migration and metastasis [19]. A dominant role is played by miRNA-29 family in regulating extracellular matrix genes such as secreted protein, acidic, Sparc, PTEN, LAMA2, collagens, integrin β, Mmp2 (Additional file 1). In addition, miRNA-125b is ubiquitously expressed in miRNAs and aberrantly expressed in a great variety of tumours [20, 21]. In some tumours, e.g. hematopoietic tumours and glioblastomas, miRNA-125b is up-regulated and displays oncogenic potential, as it induces cell growth and proliferation [22, 23]. Conversely, in other tumour types, e.g. hepatocellular carcinoma and bladder cancer, miRNA-125b is heavily down-regulated and has been reported to function as tumour-suppressor gene [24, 25]. This down-regulation is accompanied by de-repression of cellular proliferation and anti-apoptotic programs, contributing to malignant transformation. To date, several direct targets of miRNA-125b, including p53, Bcl-w, PIK3CD, c-Jun, E2F3, EPO and IL-6R and so on (Table 3), have been identified. This results in either oncogenic or tumour suppressive modes of action which contribute to either stimulate or inhibit carcinogenesis. All through the study, we noticed that levels of miRNA-26b in NPC patients treated with CCRT tended to be lower. miRNA-26b was reported as down regulated in serial cancer types including nasopharyngeal carcinoma, breast cancer, hepatocellular carcinoma, primary squamous cell lung carcinoma, squamous cell carcinoma of tongue and glioma cancers [26–30]. Therefore they were regarded as tumour suppressor miRNAs. Functional studies have identified several key targets of miRNA-26a such as PTEN, Smad1, CDK8, ATM, COX-2, TAK1,TAB3,etc. Moreover, the overexpression of the miRNA-26a in vitro leads to inhibition of cells growth by increasing apoptosis and decreasing cells proliferation (Additional file 1).

This data suggests a potential role for these circulating miRNA levels that should be explored in prospective studies. Recent studies have focused on circulating miRNAs, which have recently been reported to serve as an effective and non-invasive biomarker for detecting various cancers [31–33]. The sensitivity and specificity of circulating miRNA biomarkers for NPC are good in comparison with the serum biomarkers currently used. Yet, there is still a long way to go before circulating miRNAs could be used as a clinical diagnostic tool to provide treatment for NPC. Future studies about circulating miRNA biomarkers may focus on combining the expression profiles of circulating miRNAs from all common diseases to obtain specific biomarkers for unique disease detection.

Nevertheless, there are several limitations in this study. First, the relatively small samples of patients included make it difficult to draw definitive conclusions from our findings. Therefore, further validations of these markers in larger cohorts and in independent studies are necessary. Secondly, the method of qPCR by relative quantification approach is less accurate if measured with low levels of miRNAs, in which they may not fall into the linear range of the assay. Based on the Ct values of all samples, we believe that miRNA-29a and miRNA-125b are not in low abundance in plasma.Yet, an absolute quantification approach with standard curve calibration would be preferable for further validation of our approach. Moreover, the experimental data about six miRNAs,miRNA-22, miRNA-30c, miRNA-550, miRNA-665, miRNA-491-3p and let-7c were not present in the relevant article, but it is uncertain whether these miRNAs are specific for NPC therapy-related miRNAs. Thus, additional studies will be needed in the future. Our work, even though of interest in providing evidence for the potential value of serum miRNAs as diagnostic and prognostic biomarkers in NPC treated with concurrent chemo-radiotherapy, is rather preliminary at this stage. Detailed characterization of the miRNAs concerned is required through the addition of further experimental data in order to better understand the functional role and significance of this relationship.

## Conclusion

Different expressions of miRNAs in serum of NPC patients treated with CCRT have been reported in this study. miRNA-29a, miRNA-125b and miRNA-26b have shown reasonable sensitivity in NPC patients treated with CCRT. Their presence is confirmed in fecal occult blood test. For the first time to our knowledge, our work has also highlighted the clinical relevance of serum miRNAs in NPC treated with CCRT by demonstrating that down-expression levels of miRNAs may represent a marker of poor prognosis. This research may be considered useful as a noninvasive screening test for NPC treatment- if validated in future larger scale studies.

## Supplementary information

**Additional file 1.**

## Data Availability

The data sets used and/or analysed during the current study are available from the corresponding author on reasonable request. The data sets supporting the conclusions of this article are included within the article and its additional file.

## Abbreviations

AJCC: American Joint Committee on Cancer
AML: Acute myeloid leukemia
B-CLL: B cell chronic lymphocytic leukemia
CCRT: Concurrent chemoradiotherapy
CG: Control group
CI: Confidence interval
Ct: Threshold cycle
DFS: Disease-free survival
EG: Experimental group
HR: Hazard ratio
IMRT: Intensity-modulated radiotherapy
LNA: Locked nucleic acid
miRNA: MicroRNA
NDP: Nedaplatin
NPC: Nasopharyngeal carcinoma
OS: Overall survival
PCA: Principal component analysis
PFS: Progression-free survival
qRT-PCR: Quantitative RT-PCR
SAM: Significance analysis of microarrays
