# Supplementary material for: Serum miRNAs, a potential prognosis marker of loco-regionally advanced nasopharyngeal carcinoma patients treated with CCRT
Source: BMC Cancer. 2020 Mar 4;20:183. doi: 10.1186/s12885-020-6689-7 (PMC7057605; doi:10.1186/s12885-020-6689-7)
Supplement: Supplementary file 1 — Additional file 1. [file 12885_2020_6689_MOESM1_ESM.docx]

| **MicroRNA** | **Cancer type** | **Proved targets** | **Molecular mechansims** | **Reference** |
| --- | --- | --- | --- | --- |
| **MiR-125b**  **MiR-125b**  **MiR-125b** | Breast Cancer | KIAA1522 | miR-125b-5p inhibits breast cancer cell proliferation, migration and invasion by targeting KIAA1522. | Yongzhen et al(2018) |
|  |  | TP53INP1 | StarD13 3'-untranslated region functions as a ceRNA for TP53INP1 in prohibiting migration and invasion of breast cancer cells by regulating miR-125b activity. | Zheng et al(2018) |
|  |  | Ets-1 | Microenvironment Stimuli HGF and Hypoxia Differently Affected miR-125b and Ets-1 Function with Opposite Effects on the Invasiveness of Bone Metastatic Cells: A Comparison with Breast Carcinoma Cells. | Matteucci et al(2018) |
|  |  | Sema4C | MiR-125b regulates epithelial-mesenchymal transition via targeting Sema4C in paclitaxel-resistant breast cancer cells. | Yang et al(2015) |
|  |  | Mcl-1 | Downregulated miR-125b promotes myeloid cell leukemia-1 (Mcl-1) expression, and plays an important role in doxorubicin resistance and therapy in human breast cancer. | Xie et al(2015) |
|  | Lung Cancer | E2F2 | Circular RNA circPVT1 Promotes Proliferation and Invasion Through Sponging miR-125b and Activating E2F2 Signaling in Non-Small Cell Lung Cancer. | Li et al(2018) |
|  |  | IGFBP3 | Targeting insulin-like growth factor-binding protein-3 by microRNA-125b promotes tumor invasion and poor outcomes in non-small-cell lung cancer. | Wang et al(2017) |
|  |  | KLC2 | High expression of kinesin light chain-2, a novel target of miR-125b is associated with poor clinical outcome of elderly non-small-cell lung cancer patients. | Wang et al(2015) |
|  |  | HER-2 | proto-oncogene protein HER-2 is directly regulated by microRNAs miR-125a and miR-125b in small cell lung cancer. | Yagishita et al(2015) |
|  | Esophageal Carcinoma | BMF | MicroRNA-125b inhibits cell proliferation and induces cell apoptosis in esophageal squamous cell carcinoma by targeting BMF. | Fan et al(2018) |
|  | Gastric Cancer | MCL1 | miR-125b Suppresses Proliferation and Invasion by Targeting MCL1 in Gastric Cancer. | Wu et al(2015) |
|  |  | PPP1CA | MiR-125b promotes cell migration and invasion by targeting PPP1CA-Rb signal pathways in gastric cancer, resulting in a poor prognosis. | Wu et al(2015) |
|  | Colorectal Cancer | APC | miR-125b played an important role in triggering tumor invasion through activation of the Wnt/β-catenin signal pathway by targeting APC gene in colorectal cancer . | Yu et al(2017) |
|  |  | Gab2 | miR-125b mediates PAR2-induced cancer cell migration by targeting Gab2 in colorectal cancer. | Yang et al(2015) |
|  | Gallbladder Cancer | Bcl2 | miR-125b-5p enhances chemotherapy sensitivity to cisplatin by down-regulating Bcl2 in gallbladder cancer. | Yang et al(2017) |
|  | Cholangiocarcino-ma | IL-6R | The let-7c/miR-99a/miR-125b cluster controlled tumorigenesis by targeting IL-6, IL-6R and IGF1R. | Lin et al(2016) |
|  | Hepatocellular Carcinoma | Angpt2 | miR-125b and miR-100 negatively regulate Angpt2 expression through different mechanisms, in turn inhibit VETC formation, and consequently abrogate the VETC-dependent metastasis of hepatoma cells. | Zhou et al(2016) |
|  |  | HOTTIP | Long non-coding RNA HOTTIP is frequently up-regulated in hepatocellular carcinoma and is targeted by tumour suppressive miR-125b. | Tsang et al(2015) |
|  |  | TAZ | miR-125b may be involved in the tumorigenesis of HCC at least in part by the suppression of TAZ. | Li et al(2015) |
|  | Ovarian Cancer | SET | MicroRNA-125b Suppresses Ovarian Cancer Progression via Suppression of the Epithelial-Mesenchymal Transition Pathway by Targeting the SET Protein. | Ying et al(2016) |
|  |  | EIF4EBP1 | MicroRNAs 125a and 125b inhibit ovarian cancer cells through post-transcriptional inactivation of EIF4EBP1. | Lee et al(2016) |
|  |  | PPARγ | PPARγ inhibits ovarian cancer cells proliferation through upregulation of miR-125b. | Luo et al(2015) |
|  | Oral Cancer | STAT3 | Long non-coding RNA MALAT1 promotes oral squamous cell carcinoma development via microRNA-125b/STAT3 axis. | Chang et al(2018) |
|  | Laryngeal Carcinom | HK-2 | MiR-125b-5p suppressed the glycolysis of laryngeal squamous cell carcinoma by down-regulating hexokinase-2. | Hui et al(2018) |
|  | Thyroid Cancer | PIK3CD | MiR-125b inhibits anaplastic thyroid cancer cell migration and invasion by targeting PIK3CD. | Bu et al(2017) |
|  | Nasopharyngeal Carcinoma | A20 | MiR-125b regulates proliferation and apoptosis of nasopharyngeal carcinoma by targeting A20/NF-kB signaling pathway. | Zheng et al(2017) |
|  |  | Bcl-2 | MicroRNA-125b reverses the multidrug resistance of nasopharyngeal carcinoma cells via targeting of Bcl-2. | Yuan et al(2017) |
|  |  | p53 | Has-miR-125a and 125b are induced by treatment with cisplatin in nasopharyngeal carcinoma and inhibit apoptosis in a p53-dependent manner by targeting p53 mRNA. | Chen et al(2015) |
|  | Leukemia | A20 | MiR-125b regulates differentiation and metabolic reprogramming of T cell acute lymphoblastic leukemia by directly targeting A20. | Liu et al(2016) |
|  |  | BTG2,  MAP3K11, RPS6KA1 and PRDM1 | BTG2, MAP3K11, RPS6KA1 and PRDM1 as putative targets of microRNA miR-125b involved in acute promyelocytic leukemia cell proliferation. | Zhang et al(2016) |
|  |  | C/EBPα | The deregulated expression of miR-125b in acute myeloid leukemia is dependent on the transcription factor C/EBPα. | Romero et al(2015) |
|  | Glioblastoma | FZD6 | A regulatory circuit of miR-125b/miR-20b and Wnt signalling controls glioblastoma phenotypes through FZD6-modulated pathways. | Huang et al(2016) |
|  | Osteosarcoma | HK-2 | miR-125b suppresses the aerobic glycolysis of osteosarcoma HOS cells by downregulating the expression of hexokinase-2. | Wu et al(2017) |
|  | Chondrosarcoma | STAT3 | Results showed that MiR-125b expression was downregulated in human metastatic chondrosarcoma tissues and cells and found STAT3 as its direct target. | Bao et al(2016) |
| **MiR-22**  **MiR-22**  **MiR-22**  **MiR-22** | Gastric Cancer | NLRP3 | MiR-22 sustains NLRP3 expression and attenuates H. pylori-induced gastric carcinogenesis. | Li et al(2018) |
|  |  | NTRK2 | miR-22 was the reverse upstream regulator of NTRK2,as its upregulation downregulated NTRK2 gene and TrkB protein in GC cells. | Hu et al(2016) |
|  |  | MMP14,Snail | MicroRNA-22 inhibits tumor growth and metastasis in gastric cancer by directly targeting MMP14 and Snail. | Zuo et al(2015) |
|  |  | MTDH | MiR-22 acts as a metastasis suppressor by targeting metadherin in gastric cancer. | Tang et al(2015) |
|  | Breast Cancer | NRAS | MicroRNA-22 Suppresses Breast Cancer Cell Growth and Increases Paclitaxel Sensitivity by Targeting NRAS. | Song et al(2018) |
|  |  | NK1R-Tr ,ERα | MiR-22 downregulated the expression of NK1R-Truncated and ER-alpha to delay and weaken phosphorylation of ERK1/2 to inhibit proliferation and metastasis of breast cancer cells. | Liu et al(2019) |
|  |  | MDC1 | MiR-22-mediated downregulation of MDC1 suppresses DSB repair in differentiated MCF-7 cells. | Lee et al(2017) |
|  |  | Sirt1 | miR-22 suppresses tumorigenesis and improves radiosensitivity of breast cancer cells by targeting Sirt1. | Zhang et al(2017) |
|  |  | Sirt1,ELOVL6 | miR-22 represses de novo fatty acid synthesis and elongation by targeting ACLY and long-chain fatty acid ELOVL6 in breast cancer cells. | Koufaris et al(2016) |
|  |  | GLUT1 | MiR-22 as a prognostic factor targets glucose transporter protein type 1 in breast cancer. | Chen et al (2015) |
|  | Nasopharyngeal Carcinoma | LEF1 | miR-22 could bind to the 3'UTR of LEF1 to inhibit its expression and reverse the effect of H19 on NPCs. | Wang et al(2018) |
|  | Ovarian Cancer | CDK6, MDM2, LEF1,MYB and FOS | several key genes (CDK6, MDM2, LEF1,MYB and FOS) that participated in different pathways were involved in the mechanism of CCOC with overexpressed miR-22. | Zhen et al(2016) |
|  |  | Notch | MiR-22 Directly Suppressed the Notch Signal in Human OVCAR-3 and SKOV3 Cells. | Li et al(2016) |
|  | Cervical Cancer | eIF4EBP3 | MiR-22-3p Regulates Cell Proliferation and Inhibits Cell Apoptosis through Targeting the eIF4EBP3 Gene in Human Cervical Squamous Carcinoma Cells. | Lv et al(2018) |
|  | Colorectal Cancer | HuR | The Jun/miR-22/HuR regulatory axis contributes to tumourigenesis in colorectal cancer. | Liu et al(2018) |
|  |  | Sp1 | MicroRNA-22 suppresses the growth, migration and invasion of colorectal cancer cells through a Sp1 negative feedback loop. | Xia et al(2017) |
|  |  | TIAM1 | miR-22 and TIAM1 might play a regulatory role in the occurrence and development of colorectal cancer which were consistent with the survival curve analysis results. | Li et al(2017) |
|  |  | BTG1 | miR-22 may function as an important switch between autophagy and apoptosis to regulate 5-FU sensitivity through post-transcriptional silencing of BTG1 in colorectal cancer cells. | Zhang et al(2015) |
|  | Retinoblastoma | ENO1 | miR-22-3p inhibits the proliferation of retinoblastoma cells by negatively regulating the expression of ENO1. | Liu et al(2018) |
|  | Leukaemia | CRCT1, MYCBP and FLT3 | The inactivation of miR-22 results in the de-repression of its critical oncogenic targets such as CRCT1, MYCBP and FLT3, and thereby the activation of both CREB and MYC signalling pathways, leading to cell transformation and leukemogenesis. | Jiang et al(2016) |
|  |  | MECOM(EVI1) | miR-22 promoted monocyte/macrophage differentiation, and MECOM(EVI1) mRNA is a direct target of miR-22 and MECOM (EVI1) functions as a negative regulator in the differentiation. | Shen et al(2016) |
|  | Hepatocellular Carcinoma | CCNA2 | Waltonitone inhibits proliferation of hepatoma cells and tumorigenesis via FXR-miR-22-CCNA2 signaling pathway. | Yang et al(2016) |
|  |  | Galectin-1 | The immunosuppressive microenvironment promoted by hepatic stellate cell-derived galectin-1 in hepatocellular carcinoma can be inhibited by miR-22. | You et al(2016) |
|  |  | YWHAZ | miR-22 targets YWHAZ to inhibit metastasis of hepatocellular carcinoma and its down-regulation predicts a poor survival. | Chen et al(2016) |
|  |  | MTHFR | miR-22-3p/miR-149-5p exert different post-transcriptional effects on MTHFR under conditions of folic acid deficiency in normal and cancerous human hepatocytes. | Li et al(2017) |
|  |  | PPM1K | miR-22 regulates human but not mouse PPM1K expression,which plays an essential role in mitochondrial regulation and cell survival. | Pan et al(2015) |
|  |  | Gal-9 | the effect of the miR-22/Gal-9 axis on lymphocyte exhaustion and apoptosis, as well as tumor cell proliferation and immune evasion. | Yang et al(2015) |
|  | Osteosarcoma | S100A11 | miR-22 overexpression sensitizes MG-63 cells to cisplatin treatment and decreases the levels of S100A11. | Zhou et al(2018) |
|  |  | MDC1 | miR-22 inhibits DDR through the downregulation of MDC1 in U2OS cells after IR exposure. | Lee et al(2015) |
|  | Ewing Sarcoma | KDM3A | The histone demethylase KDM3A is a microRNA-22-regulated tumor promoter in Ewing Sarcoma. | Parrish et al(2015) |
|  | Bladder Cancer | NET1 | MiR-22-3p enhances multi‑chemoresistance by targeting NET1 in bladder cancer cells. | Xiao et al(2018) |
|  | Lung Cancer | Snail | MiR-22 inhibits lung cancer cell EMT and invasion through targeting Snail. | Zhang et al(2017) |
|  |  | ErbB3 | Hsa_circ_0012673 promotes LAC proliferation by suppressing miR-22, which targets ErbB3. | Wang et al(2018) |
|  | Melanoma | MALAT1,MMP14 and Snail | MMP14 and Snail are the functional targets of miR-22 that affect invasive and migratory abilities of melanoma cells. | Luan et al(2016) |
|  | Prostate Cancer | E-cadherin | the MTA1/Epi-miR-22/E-cadherin axis as a new epigenetic signaling pathway that promotes tumor invasion in prostate cancer. | Dhar et al(2017) |
|  |  | PTEN | hsa-miR-22 as an oncomiR contributed to prostate tumorigenesis by modulations in PI3K/AKT signaling pathways by targeting PTEN. | Budd et al(2015) |
|  |  | LAMC1 | AR stimulated miR-22 trigger decreased expression of LAMC1 protein reducing cell migration and viability and enhancing apoptosis in Prostate Cancer. | Pasqualini et al(2015) |
|  | Renal Cell Carcinoma | PTEN | MicroRNA-22 is downregulated in clear cell renal cell carcinoma, and inhibits cell growth, migration and invasion by targeting PTEN. | Fan et al(2016) |
|  |  | SIRT1 | MicroRNA-22 functions as a tumor suppressor by targeting SIRT1 in renal cell carcinoma. | Zhang et al(2016) |
|  | Glioblastoma | TIMP2 | Promotion of astrocytoma cell invasion by micro RNA-22 targeting of tissue inhibitor of matrix metalloproteinase-2. | Ohnishi et al(2017) |
|  |  | SIRT1 | miR-22 inhibits the proliferation, motility, and invasion of human glioblastoma cells by directly targeting SIRT1. | Chen et al(2016) |
|  | Tongue Carcinoma | CD147 | miR-22 regulates cell invasion, migration and proliferation in vitro through inhibiting CD147 expression in tongue squamous cell carcinoma. | Qiu et al(2016) |
|  | Neuroblastoma | BDNF | PFOS significantly increased the relative expression of miR-22, which may repress the translation of BDNF mRNA at the posttranscriptional level. | Li et al(2015) |
|  | MDS | TET2 | miR-22 transgenic mice developed myeloid malignancies associated to reduced expression of TET2, while miR-22 over expression in adult MDS was correlated with poor survival, reduced levels of global 5hmC and cellular transformation. | Coutinho et al(2015) |
| **MiR-29a**  **MiR-29a**  **MiR-29a**  **MiR-29a** | Glioblastoma | PTEN, EphB3 and SOX4 | miR-29a downregulates PTEN, EphB3 and SOX4 expression to activate a complex post-transcriptional program of growth and invasion in glioblastoma. | Zhao et al(2019) |
|  |  | Gab1 | MicroRNA-29a-3p Downregulation Causes Gab1 Upregulation to Promote Glioma Cell Proliferation. | Shao et al(2018) |
|  |  | QKI-6 | Overexpression of miR-29a reduces the oncogenic properties of glioblastoma stem cells by downregulating Quakin ene isoform 6(QKI-6). | Xi et al(2017) |
|  |  | SCAP/SREBP-1 | miR-29 transfection inhibits GBM growth in vivo via suppression of SCAP/SREBP-1. | Ru et al(2016) |
|  |  | CDC42 | miR-29a/b/c function as invasion suppressors for gliomas by targeting CDC42 and predict the prognosis of patients. | Shi et al(2017) |
|  |  | VASH2 | MiR-29a suppresses glioma-induced angiogenesis in vitro by suppressing expression of angiogenic gene VASH2. | Jia et al(2016) |
|  | Pancreatic Cancer | CAV2 | MiR-29a, targeting caveolin 2 expression, is responsible for limitation of pancreatic cancer metastasis in patients with normal level of serum CA125. | Liang et al(2018) |
|  | Gastric Cancer  Gastric Cancer | A20 | The miR-29a-3p may act as a tumor promotive miRNA by regulating cells migration through directly targeting of A20 gene in human gastric epithelial cells infected with H.pylori. | Sun et al(2018) |
|  |  | HAS3 | miR-29a-3p represses proliferation and metastasis of gastric cancer cells via attenuating HAS3 levels. | Bai et al(2018) |
|  |  | HDAC4 | lncRNA-MIAT regulates cell biological behaviors in gastric cancer through a mechanism involving the miR-29a-3p/HDAC4 axis. | Li et al(2017) |
|  |  | ITGB1 | hTERT mediates gastric cancer metastasis partially through the indirect targeting of ITGB1 by microRNA-29a. | He et al(2016) |
|  |  | VEGF | By simulating the tumor microenvironment, the MV-delivered miR-29a/c significantly suppresses VEGF expression in GC cells, inhibiting vascular cell growth, metastasis, and tube formation. | Zhang et al(2016) |
|  |  | Robo1 | MicroRNA-29a inhibits cell migration and invasion via targeting Roundabout homolog 1 in gastric cancer cells. | Liu et al(2015) |
|  |  | CDK2, CDK4, and CDK6 | Decreased miR-29a-3p expression promoted gastric cancer cell proliferation via reducing the expression of cell-cycle regulators including: CDK2, CDK4, and CDK6. | Zhao et al(2015) |
|  |  | AKT2 | MicroRNA-29s could target AKT2 to inhibit gastric cancer cells invasion ability. | Zhang et al(2015) |
|  | Lung Cancer | NRAS | MicroRNA-29a Functions as a Tumor Suppressor and Increases Cisplatin Sensitivity by Targeting NRAS in Lung Cancer. | Liu et al(2018) |
|  |  | DNMT1, DNMT3b, COL1A1, AKT1 and AKT2 | Common targets of miR-29a and miR-185 such as DNMT1, DNMT3b, COL1A1, AKT1 and AKT2 were measured. | Bibaki et al(2018) |
|  |  | LOXL2 and SERPINH1 | Downregulation of miR-29a caused overexpression of LOXL2 and SERPINH1 in lung cancer and IPF, suggesting that these genes are involved in the pathogenesis of these two diseases. | Kamikawaji et al(2016) |
|  | Hepatocellular Carcinoma | IFITM3 | miR-29a suppresses the growth and metastasis of hepatocellular carcinoma through IFITM3. | Liang et al(2018) |
|  |  | SIRT1 | MiR-29a suppresses cell proliferation by targeting SIRT1 in hepatocellular carcinoma. | Zhang et al(2018) |
|  |  | SMARCE1 | miR-29a promotes hepatitis B virus replication and expression by targeting SMARCE1 in hepatoma carcinoma. | Wu et al(2017) |
|  |  | TET1,TET3 | miR-29a promotes HCC metastasis through SOCS1-expression repression and 5-hmC modulation by directly targeting TET-family members. | Chen et al(2017) |
|  |  | CLDN1 | miR-29a suppresses growth and migration of hepatocellular carcinoma by regulating CLDN1. | Mahati et al(2017) |
|  |  | CYP2C19 | MicroRNA hsa-miR-29a-3p modulates CYP2C19 in human liver cells. | Yu et al(2015) |
|  | Colorectal Cancer | TNFAIP3 | LIFR-AS1 inhibits miR-29a expression and up-regulates downstream target TNFAIP3 expression, finally modulating the resistance of colorectal cancer to pohotodynamic therapy. | Liu et al(2018) |
|  |  | CDC42BPA | Inhibition of cell migration and invasion by miR-29a-3p in a colorectal cancer cell line through suppression of CDC42BPA mRNA expression. | He et al(2017) |
|  |  | PTEN | MiR-29a Regulates Radiosensitivity in Human Intestinal Cells by Targeting PTEN Gene. | Wang et al(2016) |
|  | Nasopharyngeal Carcinoma | STAT3,Bcl-2 | MiR-29a up-regulation decreases Taxol resistance of nasopharyngeal carcinoma CNE-1 cells possibly via inhibiting STAT3 and Bcl-2 expression. | Gao et al(2018) |
|  | Melanoma | BMI1 | MicroRNA-29a Inhibits Growth, Migration and Invasion of Melanoma A375 Cells in Vitro by Directly Targeting BMI1. | Xiong et al(2018) |
|  | Schwannoma | BMI1 | MicroRNA-29a inhibits proliferation and motility of schwannoma cells by targeting CDK6. | Ma et al(2018) |
|  | Retinoblastoma | STAT3 | miR-29a inhibits human retinoblastoma progression by targeting STAT3. | Liu et al(2018) |
|  | Leukemia | TET2 | the up-regulation of miR-29a-3p observed in CML LSCs, led to the down-regulation of the target TET2 and conferred TKI-resistance to CML LSCs in vitro. | Salati et al(2017) |
|  |  | FoxM1 | MiR-29 restrains K562 cell growth and proliferation and induces apoptosis by down-regulating FoxM1. | Wang et al(2015) |
|  |  | TETs, DNMTs, | miR‐29a might contribute to the molecular pathophysiology of T‐ALL through several newly identified targets, as well as previously identified targets (described in other neoplasias). | Oliveira et al(2015) |
|  | Breast Cancer | IGF-1R,CDC42,p85α | miR-29a-induced cell proliferation and metastasis acceleration occurred mainly through ERK phosphorylation and that IGF-1R is the upstream target gene of miR-29a, while CDC42 and p85α are the downstream target genes of miR-29a. | Li et al(2017) |
|  |  | FSCN1 | XIAP 3'-untranslated region as a ceRNA promotes FSCN1 function in inducing the progression of breast cancer by binding endogenous miR-29a-5p. | Wu et al(2017) |
|  |  | TNFR1 | miR-29a suppresses MCF-7 cell growth by downregulating tumor necrosis factor receptor 1. | Zhao et al(2017) |
|  |  | PTEN,GSK3β | PTEN and GSK3β are targeted by miR-29a, and miR-29a may contribute to ADR resistance through inhibition of the PTEN/AKT/GSK3β pathway in breast cancer cells. | Shen et al(2016) |
|  |  | HSPs | Knockdown of microRNA-29a changes the expression of heat shock proteins in breast carcinoma MCF-7 cells. | Choghaei et al(2016) |
|  |  | Robo1 | MicroRNA-29a inhibits cell migration and invasion by targeting Roundabout 1 in breast cancer cells. | Li et al(2015) |
|  | Pancreatic Cancer | TFEB,ATG9A | miR-29a inhibits PDAC autophagy by downregulation of TFEB and ATG9A. | Kwon et al(2016) |
|  |  | TTP | MicroRNA-29a Promotes Pancreatic Cancer Growth by Inhibiting Tristetraprolin. | Sun et al(2015) |
|  |  | COL1A1,  COL3A1, LAMC1 | TGF-β1 acts as a negative regulator of miR-29 in PSCs, while simultaneously upregulating ECM proteins: collagens, laminin, and fibronectin. | Kwon et al(2015) |
|  | Renal Cell Carcinoma | CDC42 | Androgen receptor (AR) promotes clear cell renal cell carcinoma (ccRCC) migration and invasion via altering the circHIAT1/miR-195-5p/29a-3p/29c-3p/**CDC42** signals. | Wang et al(2017) |
|  | Prostate Cancer | LOXL2 | Regulation of metastasis-promoting LOXL2 gene expression by antitumor microRNAs in prostate cancer. | Kato et al(2015) |
|  |  | MCL1 | MCL1,as direct targets of miR-29a,involves in inhibiting pivotal oncogenic pathways to increase apoptosis in PCa cells. | Pasqualini et al(2015) |
|  | Thyroid Carcinoma | AKT3 | miR-29a suppresses growth and metastasis in papillary thyroid carcinoma by targeting AKT3. | Li et al(2016) |
|  | Multiple Myeloma | c-Myc | MiRNA-29a as a tumor suppressor mediates PRIMA-1^Met^-induced anti-myeloma activity by targeting c-Myc. | Saha et al(2016) |
|  | Osteosarcoma | IGF,VEGF | IGF1-3’UTR can function as a ceRNA of VEGF and regulate VEGF expression by competitively binding with miR-29 family in osteosarcoma cells. | Gao et al(2016) |
|  | Nasopharyngeal Carcinoma | SPARC,  COL3A1 | miR-29a/b enhances cell migration and invasion in nasopharyngeal carcinoma progression by regulating SPARC and COL3A1 gene expression. | Qiu et al(2015) |
|  | Esophageal Carcinoma | Nfia | MiR-29a activates Hes1 by targeting Nfia in esophageal carcinoma cell line TE-1. | Liu et al(2015) |
| **MiR-29b**  **MiR-29b**  **MiR-29b**  **MiR-29b** | Leukemia | Myc,  Akt2,  CCND2 | miR-29b-1 targeting of AML1-ETO results in decreased BrdU incorporation and increased apoptosis, as well as changes in expression of genes associated with cell growth (e.g., Akt2) ,cell cycle progression (e.g., CCND2) and cellular processes (e.g., Myc).（ Myc，Akt2,CCND2） | Zaidi et al(2017) |
|  |  | T-bet,EOMES | upregulation of miR-29b in the NK cells of leukemic mice, which downregulates expression of 2 transcription factors that are critical for NK cell maturation of AML: T-bet and EOMES. | Mundy-Bosse et al(2016) |
|  | MDS | MCL-1 | Reduced miR-29b expression in MDS bone marrow cellsmight trigger transformation to OL via overexpression of MCL-1 in blastic cells. | Kirimura et al(2016) |
|  | Multiple Myeloma | FOXP1 | miR-29b inhibits the progression of multiple myeloma through downregulating FOXP1. | Wang et al(2019) |
|  |  | HDAC4 | miR-29b directly targets HDAC4 in multiple myeloma cells and its reduced mRNA expression inversely correlates with HDAC4 levels in multiple myeloma samples. | Amodio et al(2016) |
|  |  | NF-kB | genistein inhibits the proliferation and induces the apoptosis of human MM cells through  suppressing NF-kB via upregulation of miR-29b. | Xie et al(2016) |
|  |  | PSME4 | miR-29b represses PSME4 to inhibit proteasomes in multiple myeloma cells. | Jagannathan et al(2015) |
|  | Pancreatic Cancer | VEGFA | Linc00511 acts as a competing endogenous RNA to regulate VEGFA expression through sponging hsa-miR-29b-3p in pancreatic ductal adenocarcinoma. | Zhao et al(2015) |
|  | Lymphoma | BRD4 | Diminished microRNA-29b level is associated with BRD4-mediated activation of oncogenes in cutaneous T-cell lymphoma. | Kohnken et al(2018) |
|  | Neuroblastoma | P85α | In differentiating SH-SY5Y cells, expression of miR-29b downregulates expression of P85α,which results in upregulation of miR-145 and downregulation of RTF proteins. | Jauhari et al(2018) |
|  | Melanoma | LAMC1,PPIC | miR-29b as a regulator of cellular phenotype in melanoma through proteins including LAMC1 and PPIC within a network of post-transcriptionally regulated genes. | Andrews et al(2016) |
|  | Colorectal Cancer | PGRN | LncRNA H19/miR-29b-3p/PGRN Axis Promoted Epithelial-Mesenchymal Transition of Colorectal Cancer Cells by Acting on Wnt Signaling. | Ding et al(2018) |
|  | Gastric Cancer | FBXO11,  CREBZF | three miRNAs (hsamiR-421, hsa-miR-29b-1-5p, and hsa-miR-27b-5p) with two mRNAs (FBXO11 and CREBZF) that might play an important role in the gastric cancer development from premalignant adenomas. | Kim et al(2018) |
|  |  | KDM2A | RUNX3-mediated up-regulation of miR-29b suppresses the proliferation and migration of gastric cancer cells by targeting KDM2A. | Kong et al(2016) |
|  |  | AKT2 | **MiRNA-29s** could target AKT2 to inhibit gastric cancer cells invasion ability. | Zhang et al(2015) |
|  |  | DNMT3A | Deregulation between miR-29b/c and DNMT3A is associated with epigenetic silencing of the CDH1 gene, affecting cell migration and invasion in gastric cancer. | Cui et al(2018) |
|  | Pancreatic Cancer | Cbl-b | MicroRNA-29b-2-5p inhibits cell proliferation by directly targeting Cbl-b in pancreatic ductal adenocarcinoma. | Li et al(2018) |
|  |  | DNMT3b | Downregulation of miR-29b targets DNMT3b to suppress cellular apoptosis and enhance proliferation in pancreatic cancer. | Wang et al(2015) |
|  | Glioblastoma | MYCN | MiR-29b inhibits the growth of glioma via MYCN dependent way. | Sun et al(2017) |
|  |  | CDC42 | **miR-29a/b/c** function as invasion suppressors for gliomas by targeting CDC42 and predict the prognosis of patients. | Shi et al(2017) |
|  |  | CDK6 | The identification of miR-29b-1 and miR-129-3p as key regulators of CDK6 expression provides a novel link between NTS/NTSR1 activation and glioblastoma cell proliferation. | Ouyang et al(2015) |
|  |  | BCL2L2 | miR-29b attenuates tumorigenicity and stemness maintenance in human glioblastoma multiforme by directly targeting BCL2L2. | Chung et al(2015) |
|  | Breast Cancer | CCL11,  CXCL14 | MiR-29b restoration in CAFs inhibits breast cancer cellular viability and metastasis by targeting CCL11 and CXCL14. | Liu et al(2017) |
|  |  | SPIN1 | miR-29b-1-5p by downregulating SPIN1 inhibits both WNT and Akt signaling pathways,which could contribute to the effects induced by miR-29b-1 on proliferation, self-renewal, migration, invasion and chemosensitivity in TNBC cells. | Drago-Ferrante et al(2017) |
|  |  | Akt3 | miR-29b angiogenesis/tumorigenesis in breast cancer cell by targeting Akt3 and inducing VEGF and C-myc arrest in breast cancer cells. | Li et al(2017) |
|  |  | PI3K p85α, CDC42 | miR-29b targeted **PI3K p85α and CDC42,** which consequently increased p53 level by enhancing its activation and nuclear translocation in MCF7 cells. | Zhao et al(2015) |
|  | Ovarian Cancer | SIRT1 | Mir-29b Regulates Oxidative Stress by Targeting SIRT1 in Ovarian Cancer Cells. | Hou et al(2017) |
|  |  | DNMT3A/3B | A Double-Negative Feedback Interaction between MicroRNA-29b and DNMT3A/3B Contributes to Ovarian Cancer Progression. | Teng et al(2016) |
|  |  | AKT2/3 | MicroRNA-29B regulates the Warburg effect in ovarian cancer by targeting AKT2 and AKT3. | Teng et al(2015) |
|  | Bladder Cancer | DNMT3B | H19 might function as ceRNA for miR-29b-3p and relieve the suppression for DNMT3B, which led to EMT and metastasis of bladder cancer. | Lv et al(2017) |
|  | Lung Cancer | TET1 | Enhanced MAPK signaling drives ETS1-mediated induction of miR-29b leading to downregulation of TET1 and changes in epigenetic modifications in a subset of lung SCC. | Taylor et al(2016) |
|  |  | TNFAIP3 | miR-29b induces NF-kB activity and confers resistance to extrinsic apoptosis by targeting TNFAIP3 in KRAS-Induced Non-Small Cell Lung Cancers. | Langsch et al(2016) |
|  |  | MMP-2 | The results of our pilot study indicated the diagnostic potential of miR-29b and MMP-2, an inverse association between miR26a and MMP-2 in lung carcinogenesis. | Pastuszak-Lewandosk-a et al(2016) |
|  |  | FHIT | c-Myc suppresses microRNA-29b to promote tumor aggressiveness and poor outcomes in non-small cell lung cancer by targeting FHIT. | Wu et al(2015) |
|  | Cervical Cancer | STAT3 | cisplatin suppresses cervical cancer cell movement and angiogenesis via regulating miR-29b/STAT3 axis. | Li et al(2017) |
|  |  | Mcl-1 | MicroRNA hsa-miR-29b potentiates etoposide toxicity in HeLa cells via down-regulation of Mcl-1. | Kollinerová et al(2017) |
|  | Endometrial Carcinoma | VEGFA | MicroRNA-29b Inhibits Angiogenesis by Targeting VEGFA through the MAPK/ERK and PI3K/Akt Signaling Pathways in Endometrial Carcinoma. | Chen et al(2017) |
|  |  | MMP2 | BAG3 enhances MMP2 expression by suppressing miR‑29b, thereby increasing the metastatic potential of endometrioid adenocarcinomas. | Habata et al(2015) |
|  | Prostate Cancer | LOXL2 | Aberrant expression of LOXL2 enhanced migration and invasion of PCa cells，which could be regulated by anti-tumor miRNAs (**miR-26a, miR-26b and miR-29b,et.al**.) . | Kato et al(2017) |
|  |  | AURKA, TET2,  HBP1,et.al. | miR-29b significantly induces several pathways in LNCaP cells via targeting AURKA, TET2 and HBP1,et.al. | Takayama et al(2015) |
|  | Osteosarcoma | CDK6 | MiR-29b suppresses the proliferation and migration of osteosarcoma cells by targeting CDK6. | Zhu et al(2016) |
|  | Renal Cell Carcinoma | MCL-1 | miR-29b overexpression in CD8+ T cells of renal cell carcinoma patients down-modulates MCL-1 leading to immune dysfunction. | Gigante et al(2016) |
|  |  | KIF1B | miR-29b acts as an oncomiR, promoting proliferation and invasion ability through KIF1B suppression in renal cell carcinoma. | Xu et al(2015) |
|  | Nasopharyngeal Carcinoma | SPARC,  COL3A1 | **miR-29a/b** enhances cell migration and invasion in nasopharyngeal carcinoma progression by regulating SPARC and COL3A1 gene expression. | Qiu et al(2015) |
| **MiR-143**  **MiR-143**  **MiR-143**  **MiR-143**  **MiR-143** | Breast Cancer | AKT,GSK3-α,  GSK3-β,IRS-1,  BCL2 | AKT,GSK3-α,GSK3-β,IRS-1,and BCL2 could be direct posttranscriptional targets of miR-143,which maybe related to suppression of cell proliferation and migration in Breast Cancer. | García-Vázquez et al(2019) |
|  |  | MYBL2 | MYBL2 Is Targeted by miR-143-3p and Regulates Breast Cancer Cell Proliferation and Apoptosis. | Chen et al(2018) |
|  |  | MAPK7 | MiR-143-3p inhibits the proliferation, cell migration and invasion of human breast cancer cells by modulating the expression of MAPK7. | Xia et al(2018) |
|  |  | Kras2 | miR-143 has anti- metastatic effect on breast adenocarcinoma cells by repressing the expression of Kras and its downstreaming effectors. | Tavanafar et al(2017) |
|  |  | ERK5,MAP3K7 | MicroRNA-143 inhibits cell growth by targeting ERK5 and MAP3K7 in breast cancer. | Zhou et al(2017) |
|  |  | CD44 | MicroRNA-143 targets CD44 to inhibit breast cancer progression and stem cell-like properties. | Yang et al(2016) |
|  | Laryngeal Carcinoma | k-Ras | MicroRNA-143-3p suppresses cell growth and invasion in laryngeal squamous cell carcinoma via targeting the k-Ras/Raf/MEK/ERK signaling pathway. | Zhang et al(2019) |
|  | Pancreatic Cancer | KRAS | MiRNA-143 affects the proliferation and migration of pancreatic cancer cells by exerting an impact on the expression of KRAS protein. | Xu et al(2018) |
|  | Colorectal Cancer | ITGA6, ASAP3 | MicroRNA-143-3p inhibits colorectal cancer metastases by targeting ITGA6 and ASAP3. | Guo et al(2019) |
|  |  | OECC,NF-kB,  p38 MAPK | lncRNA OECC is overexpressed in colorectal cancer and may play an oncogenic role through NF-kappaB and p38 MAPK pathway activation via miR-143-3p. | Huang et al(2018) |
|  |  | ROS | miR-143 overexpression induces high ROS generation in cells treated with oxaliplatin, which translates into a functional increase in apoptosis. | Gomes et al(2018) |
|  |  | KRAS, IGF1R, Bcl-2, HK2. | As a putative tumor suppressor, miR-143 participates in CRC development and progression by targeting KRAS, IGF1R, Bcl-2, and HK2. | Gomes et al(2018) |
|  |  | PART-1,  DNMT3A | PART-1 functioned as a ceRNA of DNMT3A,by sponging miR-143,and induced tumor progression by regulating DNMT3A. | Hu et al(2017) |
|  |  | MACC1 | the abilities of apoptosis, metastasis, and invasion in CRC tumor cells were significantly suppressed by miRNA-143 targeting MACC1. | Wang et al(2016) |
|  |  | MMP7 | MicroRNA-143 inhibits colorectal cancer cell proliferation by targeting MMP7. | Yu et al(2017) |
|  |  | Bcl-2,granzyme B | miR-143 or miR-145 overexpression increases cetuximab-mediated antibody-dependent cellular cytotoxicity in human colon cancer cells via regulating Bcl-2 expression and granzyme B activity . | Gomes et al(2016) |
|  |  | K-RAS/ERK5 | Transfection of HT-29 colon cancer cells with miR-143 decreased K-RAS and ERK5 expression | Pekow J.et al.(2015) |
|  | Myelodysplastic Syndrome/Leuke-mia | MLLT3/AF9 | miR‑143 knockdown induced a decrease in the apoptosis and promoted the proliferation of SKM‑1 cells. Moreover, miR‑143 was shown to suppress MLLT3/AF9 expression by binding to its 3'‑UTR. | Cui et al(2018) |
|  |  | DAB2 | miR-143/145 differentially regulate hematopoietic stem and progenitor activity through suppression of canonical TGFβ signaling by targeting DAB2. | Lam et al(2018) |
|  |  | BCR-ABL | miRNA143 Induces K562 Cell Apoptosis Through Downregulating BCR-ABL. | Liang et al(2016) |
|  | Osteosarcoma | FOSL2 | miR-143-3p inhibits the proliferation, migration and invasion in osteosarcoma by targeting FOSL2. | Sun et al(2018) |
|  |  | MAPK7 | MiR-143 regulates the proliferation and migration of osteosarcoma cells through targeting MAPK7. | Dong et al(2017) |
|  | Hepatocellular Carcinoma | ANGPTL8 | Inhibition of miR-143-3p amplified ANGPTL8 response to treatments (glucose, insulin, Lipopolysaccharides), suggesting that the miRNA acts to suppress ANGPTL8 expression under metabolically distorted conditions. | DiStefano et al(2019) |
|  |  | PKCε | Downregulation of microRNA-143 promotes cell proliferation by regulating PKCε in hepatocellular carcinoma cells. | Tang et al(2017) |
|  |  | MALAT1,ZEB1 | MALAT1 may regulate ZEB1 expression by sponging miR-143-3p and promotes hepatocellular carcinoma progression. | Chen et al(2017) |
|  |  | Bcl-2 | MicroRNA-143 promotes apoptosis of osteosarcoma cells by caspase-3 activation via targeting Bcl-2. | Li et al(2016) |
|  |  | TLR2 | miR-143 down-regulates TLR2 expression in hepatoma cells and inhibits hepatoma cell proliferation and invasion. | Liu et al(2015) |
|  | Gastric Cancer | GATA6 | miR-143 Inhibits Cell Proliferation of Gastric Cancer Cells Through Targeting GATA6. | Guoping et al(2018) |
|  |  | MYO6 | miR-143 and miR-145 inhibit gastric cancer cell migration and metastasis by suppressing MYO6. | Lei et al(2017) |
|  |  | AKT2 | miR-143-3p acts as a novel tumor suppressive miRNA by regulating gastric tumor growth, migration and invasion through directly targeting AKT2 gene. | Wang et al(2017) |
|  |  | DNMT3A | MiR-143 inhibits cell proliferation and invasion by targeting DNMT3A in gastric cancer. | Zhang et al(2017) |
|  | Bladder Cancer | FOXD2-AS1,  ABCC3 | Long noncoding RNA FOXD2-AS1 accelerates the gemcitabine-resistance of bladder cancer by sponging miR-143 to indirectly target ABCC3 protein expression. | An et al(2018) |
|  |  | EZH2 | Honokiol inhibits bladder tumor growth by suppressing EZH2/miR-143 axis. | Zhang et al(2015) |
|  | Ovarian Cancer | PNPO | TGF-beta1-mediated PNPO expression was at least in part through the upregulation of miR-143-3p in epithelial ovarian cancer. | Zhang et al(2017) |
|  |  | RALBP1 | Downregulation of miR-143-3p caused an increased expression of RALBP1, which may be a molecular mechanism of tumorigenesis of ovarian cancer. | Zhang et al(2016) |
|  | Cervical Cancer | ELK1 | miR‐143‐5p inhibits the migration and invasion abilities of cervical cancer cells and regulates ELK1, p‐ELK1, C‐fos, Cyclin D1, and Bcl‐2 expression in vitro. | Jin et al(2017) |
|  |  | BCL-2 | the suppressive effects of miR-143 on cervical cancer cell proliferation and promotion of apoptosis is, at least in part, through suppression of BCL-2 expression. | GóMEz-GóMEz et al(2016) |
|  | Oral Carcinoma | HK-2 | MicroRNA-143 suppresses oral squamous cell carcinoma cell growth, invasion and glucose metabolism through targeting hexokinase 2. | Sun et al(2017) |
|  | Gallbladder Cancer | HIF-1ɑ | MiR-143-5p Deficiency Triggers EMT and Metastasis by Targeting HIF-1ɑ in Gallbladder Cancer. | He et al(2017) |
|  | Prostate Cancer | KLK2 | miR-143 inhibited PCa cell proliferation and migration by inhibition of KLK2. | Chu et al(2016) |
|  |  | Bcl-2 | miR-143 Induces the Apoptosis of Prostate Cancer LNCap Cells by Suppressing Bcl-2 Expression. | Ma et al(2017) |
|  | Pancreatic Cancer | TAK1 | MiR-143 Targeting TAK1 Attenuates Pancreatic Ductal Adenocarcinoma Progression via MAPK and NF-kB Pathway In Vitro. | Huang et al(2017) |
|  | Embryonal Carcinoma | β-dystrobrevin | miR-143 controls β-dystrobrevin protein expression level and impairs proliferation of RA-treated NT2/D1 cells. | Quaranta et al(2016) |
|  | Esophageal Carcinoma | QKI-5 | MiR-143-3p functions as a tumor suppressor by regulating cell proliferation, invasion and epithelialmesenchymal transition by targeting QKI-5 in esophageal squamous cell carcinoma. | He et al(2016) |
|  |  | FAM83F | miR-143 inhibits tumor progression by targeting FAM83F in esophageal squamous cell carcinoma. | Mao et al(2016) |
|  | Glioblastoma | NUAK2 | miR-143 inhibits oncogenic traits by degrading NUAK2 in glioblastoma. | Fu et al(2016) |
|  |  | BAG3 | MiR-143 enhances the antitumor activity of shikonin by targeting BAG3 expression in human glioblastoma stem cells. | Liu et al(2015) |
| **MiR-26b**  **MiR-26b**  **MiR-26b** | Breast Cancer | EZH2 | miR-26b may regulate EZH2 expression in breast cancer and may be useful as a therapeutic target for inflammatory breast cancer and noninflammatory locally advanced breast cancer. | Ding et al(2018) |
|  |  | TRPS1 | Expression of miRNA-26b-5p and its target TRPS1 is associated with radiation exposure in post-Chernobyl breast cancer. | Wilke et al(2018) |
|  |  | ST8SIA4 | Functional roles of sialylation in breast cancer progression through miR-26a/26b targeting ST8SIA4. | Ma et al(2016) |
|  | Lung Cancer | COX-2 | MiR-26b suppresses tumor cell proliferation, migration and invasion by directly targeting COX-2 in lung cancer. | Xia et al(2015) |
|  |  | MALT1,  HMGA1 | miR-26 represses IL-6 transcription through silencing the expression of MALT1 and HMGA1, and an inverse relationship between levels of miR-26 and of HMGA1 or MALT1 transcripts in LUAD), which is linked to LUAD patient survival. | Chen et al(2016) |
|  |  | PTEN | Down-regulation of microRNA-26b modulates non-small cell lung cancer cells chemoresistance and migration through the association of PTEN. | Liang et al(2015) |
|  | Glioblastoma | COX-2 | miR-26b Mimic Inhibits Glioma Proliferation In Vitro and In Vivo Suppressing COX-2 Expression. | Chen et al(2019) |
|  |  | Wee1 | MiR-26b reverses temozolomide resistance via targeting Wee1 in glioma cells. | Wang et al(2017) |
|  |  | Bcl-2 | miR-26 could facilitate apoptosis and inhibit proliferation/invasion of neuroglioma cells via downregulating Bcl-2 expression and potentiating-caspase-3 activity. | Li et al(2017) |
|  | Colorectal Cancer | FUT4 | miR-26b inhibit cell aggressiveness by regulating FUT4 in colorectal cancer. | Li et al(2017) |
|  |  | PTEN,WNT5A | MicroRNA 26b promotes colorectal cancer metastasis by downregulating PTEN and WNT5A. | Fan et al(2018) |
|  |  | DIP1, CREBBP, BRCA1, MDM2 | miR-26b expression was shown to be upregulated with disease progression in tissues and serum of UC and UCC patients,which might mediate cross talk of divergent pathways by targeting DIP1, CREBBP, BRCA1, and MDM2. | Benderska et al(2015) |
|  | Bladder Cancer | PDCD10 | miRNA-26a-5p and miR-26b-5p inhibit the proliferation of bladder cancer cells by regulating PDCD10. | Wu et al(2018) |
|  |  | PLOD2 | Tumour-suppressive miRNA-26a-5p and miR-26b-5p inhibit cell aggressiveness by regulating PLOD2 in bladder cancer. | Miyamoto et al(2016) |
|  | Multiple Myeloma | JAG1 | miR-26b-5p suppresses proliferation and promotes apoptosis in multiple myeloma cells by targeting JAG1. | Jia et al(2018) |
|  | Hepatocellular Carcinoma | SMAD1 | Twist1-related miR-26b-5p suppresses epithelialmesenchymal transition, migration and invasion by targeting SMAD1 in hepatocellular carcinoma. | Wang et al(2016) |
|  |  | PI3K | MicroRNA-26b inhibits the tumor growth of human liver cancer through the PI3K/Akt and NF-κB/MMP-9/VEGF pathways. | Feng et al(2018) |
|  |  | VE-cadherin, Snail,  MMP2 | miR-26b-5p could suppress vascular mimicry (VM) and angiogenesis by down-regulating the expression of VE-cadherin, Snail and MMP2 and could inhibit the apoptosis of HCC cells. | Wang et al(2016) |
|  |  | EphA2 | MicroRNA-26b Enhances the Radiosensitivity of Hepatocellular Carcinoma Cells by Targeting EphA2. | Jin et al(2016) |
|  |  | Mcl-1 | Mcl-1 Is a Novel Target of miR-26b That Is Associated with the Apoptosis Induced by TRAIL in HCC Cells. | Chunlin et al(2015) |
|  | Melanoma | TRAF5 | MiR-26b inhibits melanoma cell proliferation and enhances apoptosis by suppressing TRAF5-mediated MAPK activation. | Li et al(2016) |
|  | Gastric Cancer | KPNA2 | MicroRNA-26b inhibits tumor metastasis by targeting the KPNA2/c-jun pathway in human gastric cancer. | Tsai et al(2016) |
|  |  | HGF | miR-26a/b Inhibit Tumor Growth and Angiogenesis by Targeting the HGF-VEGF Axis in Gastric Carcinoma. | Si et al(2017) |
|  | Esophageal Carcinoma | MYC | miR-26a and miR-26b inhibit esophageal squamous cancer cell proliferation through suppression of c-MYC pathway. | Li et al(2017) |
|  | Leukemia | PIK3CD | Regulation of PI3K signaling in T-cell acute lymphoblastic leukemia: a novel PTEN/Ikaros/miR-26b mechanism reveals a critical targetable role for PIK3CD. | Yuan et al(2017) |
|  | Prostate Cancer | LOXL2 | The expression levels of LOXL2 mRNA and protein were markedly suppressed in transfected prostate cancer cells with microRNAs miR-26a, miR-26b, miR-29a, miR-29b, miR-29c and miR-218. | Kato et al(2017) |
|  |  | ULK2 | MiR-26b inhibits autophagy by targeting ULK2 in prostate cancer cells. | Clotaire et al(2016) |
|  |  | LARP1 | MicroRNA-26a/b directly regulate LARP1 and inhibit cancer cell invasion in prostate cancer. | Kato et al(2015) |
|  | Renal Cell Carcinoma | LOXL2,PLOD2 | Regulation of the collagen cross-linking enzymes LOXL2 and PLOD2 by tumor-suppressive microRNA-26a/b in renal cell carcinoma. | Kurozumi et al(2016) |
|  | Osteosarcoma | PFKFB3 | MicroRNA-26b inhibits osteosarcoma cell migration and invasion by down-regulating PFKFB3 expression. | Zheng et al(2015) |
|  |  | CTGF,Smad1 | MicroRNA-26b inhibits metastasis of osteosarcoma via targeting CTGF and Smad1. | Duan et al(2015) |
|  | Tongue Carcinoma | COX-2 | miR-26b is downregulated in human tongue squamous cell carcinoma and regulates cell proliferation and metastasis through a COX-2-dependent mechanism. | Cao et al(2015) |
|  | Oral Carcinoma | TMEM184B | Loss of tumour-suppressive miR-26a/b enhanced cancer cell migration and invasion in OSCC through direct regulation of TMEM184B | Fukumoto et al(2015) |

**参考文献：Reference**

1. Yongzhen L , Yongxia W , Hongzhe F , et al. miR-125b-5p inhibits breast cancer cell proliferation, migration and invasion by targeting KIAA1522[J]. Biochemical and Biophysical Research Communications, 2018:S0006291X18318667-.
2. Zheng L, Li X, Chou J, et al. StarD13 3’-untranslated region functions as a ceRNA for TP53INP1 in prohibiting migration and invasion of breast cancer cells by regulating miR-125b activity[J]. European journal of cell biology, 2018, 97(1): 23-31.
3. Matteucci E, Maroni P, Nicassio F, et al. Microenvironment stimuli HGF and hypoxia differently affected miR-125b and Ets-1 function with opposite effects on the invasiveness of bone metastatic cells: a comparison with breast carcinoma cells[J]. International journal of molecular sciences, 2018, 19(1): 258.
4. Yang Q, Wang Y, Lu X, et al. MiR-125b regulates epithelial-mesenchymal transition via targeting Sema4C in paclitaxel-resistant breast cancer cells[J]. Oncotarget, 2015, 6(5): 3268.
5. Xie X, Hu Y, Xu L, et al. The role of miR-125b-mitochondria-caspase-3 pathway in doxorubicin resistance and therapy in human breast cancer[J]. Tumor Biology, 2015, 36(9): 7185-7194.
6. Li X, Zhang Z, Jiang H, et al. Circular RNA circPVT1 promotes proliferation and invasion through sponging miR-125b and activating E2F2 signaling in non-small cell lung Cancer[J]. Cellular Physiology and Biochemistry, 2018, 51(5): 2324-2340.
7. Wang H H, Wang Y C, Wu D W, et al. Targeting insulin-like growth factor-binding protein-3 by microRNA-125b promotes tumor invasion and poor outcomes in non-small-cell lung cancer[J]. Tumor Biology, 2017, 39(4): 1010428317694316.
8. Wang M, Zhu X, Sha Z, et al. High expression of kinesin light chain-2, a novel target of miR-125b, is associated with poor clinical outcome of elderly non-small-cell lung cancer patients[J]. British journal of cancer, 2015, 112(5): 874.
9. Yagishita S, Fujita Y, Kitazono S, et al. Chemotherapy-Regulated microRNA-125–HER2 Pathway as a Novel Therapeutic Target for Trastuzumab-Mediated Cellular Cytotoxicity in Small Cell Lung Cancer[J]. Molecular cancer therapeutics, 2015, 14(6): 1414-1423.
10. Fan Y X, Bian X H, Qian P D, et al. MicroRNA-125b inhibits cell proliferation and induces cell apoptosis in esophageal squamous cell carcinoma by targeting BMF[J]. Oncology reports, 2018, 40(1): 61-72.
11. Wu S, Liu F, Xie L, et al. miR-125b suppresses proliferation and invasion by targeting MCL1 in gastric cancer[J]. BioMed research international, 2015, 2015:1-10.
12. Wu J G, Wang J J, Jiang X, et al. MiR-125b promotes cell migration and invasion by targeting PPP1CA-Rb signal pathways in gastric cancer, resulting in a poor prognosis[J]. Gastric Cancer, 2015, 18(4): 729-739.
13. Yu X, Shi W, Zhang Y, et al. CXCL12/CXCR4 axis induced miR-125b promotes invasion and confers 5-fluorouracil resistance through enhancing autophagy in colorectal cancer[J]. Scientific reports, 2017, 7: 42226.
14. Yang L, Ma Y, Han W, et al. Proteinase-activated receptor 2 promotes cancer cell migration through RNA methylation-mediated repression of miR-125b[J]. Journal of Biological Chemistry, 2015, 290(44): 26627-26637.
15. Yang D, Zhan M, Chen T, et al. miR-125b-5p enhances chemotherapy sensitivity to cisplatin by down-regulating Bcl2 in gallbladder cancer[J]. Scientific reports, 2017, 7: 43109.
16. Lin K Y, Ye H, Han B W, et al. Genome-wide screen identified let-7c/miR-99a/miR-125b regulating tumor progression and stem-like properties in cholangiocarcinoma[J]. Oncogene, 2016, 35(26): 3376.
17. Zhou H C, Fang J H, Shang L R, et al. MicroRNAs miR‐125b and miR‐100 suppress metastasis of hepatocellular carcinoma by disrupting the formation of vessels that encapsulate tumour clusters[J]. The Journal of pathology, 2016, 240(4): 450-460.
18. Tsang F H C, Au S L K, Wei L, et al. Long non‐coding RNA HOTTIP is frequently up‐regulated in hepatocellular carcinoma and is targeted by tumour suppressive miR‐125b[J]. Liver international, 2015, 35(5): 1597-1606.
19. Li J, Fang L, Yu W, et al. MicroRNA-125b suppresses the migration and invasion of hepatocellular carcinoma cells by targeting transcriptional coactivator with PDZ-binding motif[J]. Oncology letters, 2015, 9(4): 1971-1975.
20. Ying X, Wei K, Lin Z, et al. Microrna-125b suppresses ovarian cancer progression via suppression of the epithelial-mesenchymal transition pathway by targeting the set protein[J]. Cellular Physiology and Biochemistry, 2016, 39(2): 501-510.
21. Lee M, Kim E J, Jeon M J. MicroRNAs 125a and 125b inhibit ovarian cancer cells through post-transcriptional inactivation of EIF4EBP1[J]. Oncotarget, 2016, 7(8): 8726.
22. Luo S, Wang J, Ma Y, et al. PPARγ inhibits ovarian cancer cells proliferation through upregulation of miR-125b[J]. Biochemical and biophysical research communications, 2015, 462(2): 85-90.
23. Chang S M, Hu W W. Long non‐coding RNA MALAT1 promotes oral squamous cell carcinoma development via microRNA‐125b/STAT3 axis[J]. Journal of cellular physiology, 2018, 233(4): 3384-3396.
24. Hui L, Zhang J, Guo X. MiR-125b-5p suppressed the glycolysis of laryngeal squamous cell carcinoma by down-regulating hexokinase-2[J]. Biomedicine & Pharmacotherapy, 2018, 103: 1194-1201.
25. Bu Q, You F, Pan G, et al. MiR-125b inhibits anaplastic thyroid cancer cell migration and invasion by targeting PIK3CD[J]. Biomedicine & Pharmacotherapy, 2017, 88: 443-448.
26. Zheng Z, Qu J Q, Yi H M, et al. MiR-125b regulates proliferation and apoptosis of nasopharyngeal carcinoma by targeting A20/NF-κB signaling pathway[J]. Cell death & disease, 2017, 8(6): e2855.
27. Yuan T Z, Zhang H H, Lin X L, et al. microRNA-125b reverses the multidrug resistance of nasopharyngeal carcinoma cells via targeting of Bcl-2[J]. Molecular medicine reports, 2017, 15(4): 2223-2228.
28. Chen J J, Liu S X, Chen M Z, et al. Has‑miR‑125a and 125b are induced by treatment with cisplatin in nasopharyngeal carcinoma and inhibit apoptosis in a p53‑dependent manner by targeting p53 mRNA[J]. Molecular medicine reports, 2015, 12(3): 3569-3574.
29. Liu Z, Smith K R, Khong H T, et al. miR-125b regulates differentiation and metabolic reprogramming of T cell acute lymphoblastic leukemia by directly targeting A20[J]. Oncotarget, 2016, 7(48): 78667.
30. Zhang Y, Zeng C, Lu S, et al. Identification of miR-125b targets involved in acute promyelocytic leukemia cell proliferation[J]. Biochemical and biophysical research communications, 2016, 478(4): 1758-1763.
31. Romero P V, Cialfi S, Palermo R, et al. The deregulated expression of miR-125b in acute myeloid leukemia is dependent on the transcription factor C/EBPα[J]. Leukemia, 2015, 29(12): 2442.
32. Huang T, Alvarez A A, Pangeni R P, et al. A regulatory circuit of miR-125b/miR-20b and Wnt signalling controls glioblastoma phenotypes through FZD6-modulated pathways[J]. Nature communications, 2016, 7: 12885.
33. Wu Y, He H, Wu B, et al. miR-125b suppresses the aerobic glycolysis of osteosarcoma HOS cells by downregulating the expression of hexokinase-2[J]. Xi bao yu fen zi mian yi xue za zhi= Chinese journal of cellular and molecular immunology, 2017, 33(10): 1365-1370.
34. Bao X, Ren T, Huang Y, et al. Induction of the mesenchymal to epithelial transition by demethylation-activated microRNA-125b is involved in the anti-migration/invasion effects of arsenic trioxide on human chondrosarcoma[J]. Journal of Experimental & Clinical Cancer Research, 2016, 35(1): 129.
35. Li S, Liang X, Ma L, et al. MiR-22 sustains NLRP3 expression and attenuates H. pylori-induced gastric carcinogenesis[J]. Oncogene, 2018, 37(7): 884.
36. Hu J, Huang Y, Wu Y, et al. NTRK2 is an oncogene and associated with microRNA-22 regulation in human gastric cancer cell lines[J]. Tumor Biology, 2016, 37(11): 15115-15123.
37. Zuo Q F, Cao L Y, Yu T, et al. MicroRNA-22 inhibits tumor growth and metastasis in gastric cancer by directly targeting MMP14 and Snail[J]. Cell death & disease, 2015, 6(11): e2000.
38. Tang Y, Liu X, Su B, et al. microRNA‑22 acts as a metastasis suppressor by targeting metadherin in gastric cancer[J]. Molecular medicine reports, 2015, 11(1): 454-460.
39. Song Y K , Wang Y , Wen Y Y , et al. MicroRNA-22 Suppresses Breast Cancer Cell Growth and Increases Paclitaxel Sensitivity by Targeting NRAS[J]. Technology in Cancer Research & Treatment, 2018, 17.
40. Liu X, Zhang L, Tong Y, et al. MicroRNA-22 inhibits proliferation, invasion and metastasis of breast cancer cells through targeting truncated neurokinin-1 receptor and ERα[J]. Life sciences, 2019, 217: 57-69.
41. Lee J H , Park S J , Kim S W , et al. c-Fos-dependent miR-22 targets MDC1 and regulates DNA repair in terminally differentiated cells[J]. Oncotarget, 2017, 8(29):48204-48221.
42. Zhang X, Li Y, Wang D, et al. miR-22 suppresses tumorigenesis and improves radiosensitivity of breast cancer cells by targeting Sirt1[J]. Biological research, 2017, 50(1): 27.
43. Koufaris C, Valbuena G N, Pomyen Y, et al. Systematic integration of molecular profiles identifies miR-22 as a regulator of lipid and folate metabolism in breast cancer cells[J]. Oncogene, 2016, 35(21): 2766.
44. Chen B, Tang H, Liu X, Liu P, Yang L, Xie X, Ye F, Song C, Xie X, Wei W.miR-22 as a prognostic factor targets glucose transporter protein type 1 in breast cancer. Cancer Lett. 2015;356(2 Pt B):410-7.
45. Wang X, Zou M, Li J, et al. LncRNA H19 targets miR‐22 to modulate H2O2‐induced deregulation in nucleus pulposus cell senescence, proliferation, and ECM synthesis through Wnt signaling[J]. Journal of cellular biochemistry, 2018, 119(6): 4990-5002.
46. Zhen Y B , Guo X L , Xu B , et al. Gene expression profiling analysis of the role of miR-22 in clear cell ovarian cancer[J]. Neoplasma, 2016, 63(06):856-864.
47. Li Y, Gu Y, Tang N, et al. miR-22-Notch Signaling Pathway Is Involved in the Regulation of the Apoptosis and Autophagy in Human Ovarian Cancer Cells[J]. Biological and Pharmaceutical Bulletin, 2018, 41(8): 1237-1242.
48. Lv K, Liu Z, Feng J, et al. MiR-22-3p regulates cell proliferation and inhibits cell apoptosis through targeting the eIF4EBP3 gene in human cervical squamous carcinoma cells[J]. International journal of medical sciences, 2018, 15(2): 142.
49. Liu Y , Chen X , Cheng R , et al. The Jun/miR-22/HuR regulatory axis contributes to tumourigenesis in colorectal cancer[J]. Molecular Cancer, 2018, 17(1):11.
50. Xia S S , Zhang G J , Liu Z L , et al. MicroRNA-22 suppresses the growth, migration and invasion of colorectal cancer cells through a Sp1 negative feedback loop[J]. Oncotarget, 2017, 8(22):36266-36278.
51. Li B, Li B, Sun H, et al. The predicted target gene validation, function, and prognosis studies of miRNA-22 in colorectal cancer tissue[J]. Tumor Biology, 2017, 39(3): 1010428317692257.
52. Zhang H , Tang J , Li C , et al. MiR-22 regulates 5-FU sensitivity by inhibiting autophagy and promoting apoptosis in colorectal cancer cells[J]. Cancer Letters, 2015, 356(2):781-790.
53. Liu Y, Li H, Liu Y, et al. MiR-22-3p targeting alpha-enolase 1 regulates the proliferation of retinoblastoma cells.[J]. Biomedicine & Pharmacotherapy, 2018, 105:805-812.
54. Jiang X , Hu C , Arnovitz S , et al. miR-22 has a potent anti-tumour role with therapeutic potential in acute myeloid leukaemia[J]. Nature Communications, 2016, 7:11452.
55. Shen C, Chen M T, Zhang X H, et al. The PU. 1-modulated microRNA-22 is a regulator of monocyte/macrophage differentiation and acute myeloid leukemia[J]. PLoS genetics, 2016, 12(9): e1006259.
56. Yang F , Gong J , Wang G , et al. Waltonitone inhibits proliferation of hepatoma cells and tumorigenesis via FXR-miR-22-CCNA2 signaling pathway[J]. Oncotarget, 2016, 7(46):75165-75175.
57. You Y , Tan J X , Dai H S , et al. MiRNA-22 inhibits oncogene galectin-1 in hepatocellular carcinoma[J]. Oncotarget, 2016, 7(35):57099-57116.
58. Chen M, Hu W, Xiong C L, et al. miR-22 targets YWHAZ to inhibit metastasis of hepatocellular carcinoma and its down-regulation predicts a poor survival[J]. Oncotarget, 2016, 7(49): 80751.
59. Li C, Ni J, Liu Y X, et al. response of mirna-22-3p and mirna-149-5p to folate deficiency and the differential regulation of mthfr expression in normal and cancerous human hepatocytes[J]. PloS one, 2017, 12(1): e0168049.
60. Pan B F , Gao C , Ren S X , et al. Regulation of PP2Cm expression by miRNA-204/211 and miRNA-22 in mouse and human cells[J]. Acta Pharmacologica Sinica, 2015, 36(12):1480-1486.
61. Yang Q, Jiang W, Zhuang C, et al. microRNA-22 downregulation of galectin-9 influences lymphocyte apoptosis and tumor cell proliferation in liver cancer[J]. Oncology reports, 2015, 34(4): 1771-1778.
62. Zhou X, Natino D, Zhai X, et al. MicroRNA-22 inhibits the proliferation and migration, and increases the cisplatin sensitivity, of osteosarcoma cells[J]. Molecular medicine reports, 2018, 17(5): 7209-7217.
63. Lee J H , Park S J , Jeong S Y , et al. MicroRNA-22 Suppresses DNA Repair and Promotes Genomic Instability through Targeting of MDC1[J]. Cancer Research, 2015, 75(7):1298-1310.
64. Parrish JK, Sechler M, Winn RA, Jedlicka P.The histone demethylase KDM3A is a microRNA-22-regulated tumor promoter in Ewing Sarcoma. Oncogene. 2015 Jan 8;34(2):257-62
65. Xiao J, Niu S, Zhu J, et al. miR‑22‑3p enhances multi‑chemoresistance by targeting NET1 in bladder cancer cells[J]. Oncology reports, 2018, 39(6): 2731-2740.
66. Zhang K , Li X Y , Wang Z M , et al. MiR-22 inhibits lung cancer cell EMT and invasion through targeting Snail[J]. Eur Rev Med Pharmacol Sci, 2017:3598-3604.
67. Wang X, Zhu X, Zhang H, et al. Increased circular RNA hsa_circ_0012673 acts as a sponge of miR-22 to promote lung adenocarcinoma proliferation[J]. Biochemical and biophysical research communications, 2018, 496(4): 1069-1075.
68. Luan W, Li L, Shi Y, et al. Long non-coding RNA MALAT1 acts as a competing endogenous RNA to promote malignant melanoma growth and metastasis by sponging miR-22[J]. Oncotarget, 2016, 7(39): 63901.
69. Dhar S , Kumar A , Gomez C R , et al. MTA1-activated Epi-microRNA-22 regulates E-cadherin and prostate cancer invasiveness[J]. FEBS Letters, 2017, 591(6):924-933.
70. Budd W T, Seashols-Williams S J, Clark G C, et al. Dual action of miR-125b as a tumor suppressor and oncomiR-22 promotes prostate cancer tumorigenesis[J]. PLoS One, 2015, 10(11): e0142373.
71. Pasqualini L , Bu H , Puhr M , et al. miR-22 and miR-29a are members of the androgen receptor cistrome modulating **LAMC1** and Mcl-1 in prostate cancer[J]. Molecular Endocrinology, 2015, 29(7):1037-54.
72. Fan W, Huang J, Xiao H, et al. MicroRNA-22 is downregulated in clear cell renal cell carcinoma, and inhibits cell growth, migration and invasion by targeting PTEN[J]. Molecular medicine reports, 2016, 13(6): 4800-4806.
73. Zhang S, Zhang D, Yi C, et al. MicroRNA-22 functions as a tumor suppressor by targeting SIRT1 in renal cell carcinoma[J]. Oncology reports, 2016, 35(1): 559-567.
74. Ohnishi Y I , Iwatsuki K , Ishihara M , et al. Promotion of astrocytoma cell invasion by micro RNA-22 targeting of tissue inhibitor of matrix metalloproteinase-2.[J]. J Neurosurg Spine, 2017, 26(3):396-403.
75. Chen H, Lu Q, Fei X, et al. miR-22 inhibits the proliferation, motility, and invasion of human glioblastoma cells by directly targeting SIRT1[J]. Tumor Biology, 2016, 37(5): 6761-6768.
76. Qiu K, Huang Z, Huang Z, et al. miR-22 regulates cell invasion, migration and proliferation in vitro through inhibiting CD147 expression in tongue squamous cell carcinoma[J]. Archives of oral biology, 2016, 66: 92-97.
77. Li W , He Q Z , Wu C Q , et al. PFOS Disturbs BDNF-ERK-CREB Signalling in Association with Increased MicroRNA-22 in SH-SY5Y Cells[J]. BioMed Research International, 2015, 2015:1-10.
78. Coutinho D F, Monte-Mór B C R, Vianna D T, et al. TET2 expression level and 5-hydroxymethylcytosine are decreased in refractory cytopenia of childhood[J]. Leukemia research, 2015, 39(10): 1103-1108.
79. Zhao Y, Huang W, Kim T M, et al. MicroRNA-29a activates a multi-component growth and invasion program in glioblastoma[J]. Journal of Experimental & Clinical Cancer Research, 2019, 38(1): 36.
80. Shao N, Wang D, Wang Y, et al. MicroRNA-29a-3p Downregulation Causes Gab1 upregulation to promote glioma cell proliferation[J]. Cellular Physiology and Biochemistry, 2018, 48(2): 450-460.
81. Xi Z, Wang P, Xue Y, et al. Overexpression of miR-29a reduces the oncogenic properties of glioblastoma stem cells by downregulating Quaking gene isoform 6[J]. Oncotarget, 2017, 8(15): 24949.
82. Ru P, Hu P, Geng F, et al. Feedback loop regulation of SCAP/SREBP-1 by miR-29 modulates EGFR signaling-driven glioblastoma growth[J]. Cell reports, 2016, 16(6): 1527-1535.
83. Shi C, Ren L, Sun C, et al. miR-29a/b/c function as invasion suppressors for gliomas by targeting CDC42 and predict the prognosis of patients[J]. British journal of cancer, 2017, 117(7): 1036.
84. Jia P, Cai H, Liu X, et al. Long non-coding RNA H19 regulates glioma angiogenesis and the biological behavior of glioma-associated endothelial cells by inhibiting microRNA-29a[J]. Cancer letters, 2016, 381(2): 359-369.
85. Liang C, Shi S, Meng Q, et al. MiR‐29a, targeting caveolin 2 expression, is responsible for limitation of pancreatic cancer metastasis in patients with normal level of serum CA125[J]. International journal of cancer, 2018, 143(11): 2919-2931.
86. Sun F, Ni Y, Zhu H, et al. microRNA-29a-3p, up-regulated in human gastric cells and tissues with H. Pylori infection, promotes the migration of GES-1 cells via A20-mediated EMT pathway[J]. Cellular Physiology and Biochemistry, 2018, 51(3): 1250-1263.
87. Bai F, Jiu M, You Y, et al. miR‑29a‑3p represses proliferation and metastasis of gastric cancer cells via attenuating HAS3 levels[J]. Molecular medicine reports, 2018, 17(6): 8145-8152.
88. Li Y, Wang K, Wei Y, et al. lncRNA-MIAT regulates cell biological behaviors in gastric cancer through a mechanism involving the miR-29a-3p/HDAC4 axis[J]. Oncology reports, 2017, 38(6): 3465-3472.
89. He B, Xiao Y F, Tang B, et al. hTERT mediates gastric cancer metastasis partially through the indirect targeting of ITGB1 by microRNA-29a[J]. Scientific reports, 2016, 6: 21955.
90. Zhang H, Bai M, Deng T, et al. Cell-derived microvesicles mediate the delivery of miR-29a/c to suppress angiogenesis in gastric carcinoma[J]. Cancer letters, 2016, 375(2): 331-339.
91. Liu X, Cai J, Sun Y, et al. MicroRNA-29a inhibits cell migration and invasion via targeting Roundabout homolog 1 in gastric cancer cells[J]. Molecular medicine reports, 2015, 12(3): 3944-3950.
92. Zhao Z, Wang L, Song W, et al. Reduced miR-29a-3p expression is linked to the cell proliferation and cell migration in gastric cancer[J]. World journal of surgical oncology, 2015, 13(1): 101.
93. Zhang H, Cheng Y, Jia C, et al. MicroRNA-29s could target AKT2 to inhibit gastric cancer cells invasion ability[J]. Medical Oncology, 2015, 32(1): 342.
94. Liu X, Lv X, Yang Q, et al. MicroRNA-29a functions as a tumor suppressor and increases cisplatin sensitivity by targeting NRAS in lung cancer[J]. Technology in cancer research & treatment, 2018, 17: 1533033818758905.
95. Bibaki E, Tsitoura E, Vasarmidi E, et al. miR-185 and miR-29a are similarly expressed in the bronchoalveolar lavage cells in IPF and lung cancer but common targets DNMT1 and COL1A1 show disease specific patterns[J]. Molecular medicine reports, 2018, 17(5): 7105-7112.
96. Kamikawaji K, Seki N, Watanabe M, et al. Regulation of LOXL2 and SERPINH1 by antitumor microRNA-29a in lung cancer with idiopathic pulmonary fibrosis[J]. Journal of human genetics, 2016, 61(12): 985.
97. Liang Y, Li E, Min J, et al. miR‑29a suppresses the growth and metastasis of hepatocellular carcinoma through IFITM3[J]. Oncology reports, 2018, 40(6): 3261-3272.
98. Zhang Y, Yang L, Wang S, et al. MiR-29a suppresses cell proliferation by targeting SIRT1 in hepatocellular carcinoma[J]. Cancer Biomarkers, 2018 (Preprint): 1-9.
99. Wu H J, Zhuo Y, Zhou Y C, et al. miR-29a promotes hepatitis B virus replication and expression by targeting SMARCE1 in hepatoma carcinoma[J]. World journal of gastroenterology, 2017, 23(25): 4569.
100. Chen Q, Yin D, Zhang Y, et al. MicroRNA-29a induces loss of 5-hydroxymethylcytosine and promotes metastasis of hepatocellular carcinoma through a TET–SOCS1–MMP9 signaling axis[J]. Cell death & disease, 2017, 8(6): e2906.
101. Mahati S, Xiao L, Yang Y, et al. miR-29a suppresses growth and migration of hepatocellular carcinoma by regulating CLDN1[J]. Biochemical and biophysical research communications, 2017, 486(3): 732-737.
102. Yu D, Green B, Tolleson W H, et al. MicroRNA hsa-miR-29a-3p modulates CYP2C19 in human liver cells[J]. Biochemical pharmacology, 2015, 98(1): 215-223.
103. Liu K, Yao H, Wen Y, et al. Functional role of a long non-coding RNA LIFR-AS1/miR-29a/TNFAIP3 axis in colorectal cancer resistance to pohotodynamic therapy[J]. Biochimica et Biophysica Acta (BBA)-Molecular Basis of Disease, 2018, 1864(9): 2871-2880.
104. He P Y, Yip W K, Chai B L, et al. Inhibition of cell migration and invasion by miR‑29a‑3p in a colorectal cancer cell line through suppression of CDC42BPA mRNA expression[J]. Oncology reports, 2017, 38(6): 3554-3566.
105. Wang J, Xu J, Fu J, et al. MiR-29a regulates radiosensitivity in human intestinal cells by targeting PTEN gene[J]. Radiation research, 2016, 186(3): 292-301.
106. Gao J, Shao Z, Yan M, et al. Targeted regulation of STAT3 by miR-29a in mediating Taxol resistance of nasopharyngeal carcinoma cell line CNE-1[J]. Cancer Biomarkers, 2018 (Preprint): 1-8.
107. Xiong Y, Liu L, Qiu Y, et al. MicroRNA-29a Inhibits Growth, Migration and Invasion of Melanoma A375 Cells in Vitro by Directly Targeting BMI1[J]. Cellular Physiology and Biochemistry, 2018, 50(1): 385-397.
108. Ma J, Li T, Yuan H, et al. MicroRNA‐29a inhibits proliferation and motility of schwannoma cells by targeting CDK6[J]. Journal of cellular biochemistry, 2018, 119(3): 2617-2626.
109. Liu S, Zhang X, Hu C, et al. miR-29a inhibits human retinoblastoma progression by targeting STAT3[J]. Oncology reports, 2018, 39(2): 739-746.
110. Salati S, Salvestrini V, Carretta C, et al. Deregulated expression of miR-29a-3p, miR-494-3p and miR-660-5p affects sensitivity to tyrosine kinase inhibitors in CML leukemic stem cells[J]. Oncotarget, 2017, 8(30): 49451.
111. Wang X, Zhong H, Wang L, et al. MiR-29 induces K562 cell apoptosis by down-regulating FOXM1[J]. Medical science monitor: international medical journal of experimental and clinical research, 2015, 21: 3115.
112. Oliveira L H, Schiavinato J L, Fráguas M S, et al. Potential roles of micro RNA‐29a in the molecular pathophysiology of T‐cell acute lymphoblastic leukemia[J]. Cancer science, 2015, 106(10): 1264-1277.
113. Li Z, Xiong Q, Xu L, et al. miR-29a regulated ER-positive breast cancer cell growth and invasion and is involved in the insulin signaling pathway[J]. Oncotarget, 2017, 8(20): 32566.
114. Wu Q, Yan H, Tao S Q, et al. XIAP 3′-untranslated region as a ceRNA promotes FSCN1 function in inducing the progression of breast cancer by binding endogenous miR-29a-5p[J]. Oncotarget, 2017, 8(10): 16784.
115. Zhao Y, Yang F, Li W, et al. miR-29a suppresses MCF-7 cell growth by downregulating tumor necrosis factor receptor 1[J]. Tumor Biology, 2017, 39(2): 1010428317692264.
116. Shen H, Li L, Yang S, et al. MicroRNA-29a contributes to drug-resistance of breast cancer cells to adriamycin through PTEN/AKT/GSK3β signaling pathway[J]. Gene, 2016, 593(1): 84-90.
117. Choghaei E, Khamisipour G, Falahati M, et al. Knockdown of microRNA-29a changes the expression of heat shock proteins in breast carcinoma MCF-7 cells[J]. Oncology Research Featuring Preclinical and Clinical Cancer Therapeutics, 2016, 23(1-2): 69-78.
118. Li H, Luo J, Xu B, et al. MicroRNA‑29a inhibits cell migration and invasion by targeting Roundabout 1 in breast cancer cells[J]. Molecular medicine reports, 2015, 12(2): 3121-3126.
119. Kwon J J, Willy J A, Quirin K A, et al. Novel role of miR-29a in pancreatic cancer autophagy and its therapeutic potential[J]. Oncotarget, 2016, 7(44): 71635.
120. Sun X J, Liu B Y, Yan S, et al. MicroRNA-29a promotes pancreatic cancer growth by inhibiting tristetraprolin[J]. Cellular Physiology and Biochemistry, 2015, 37(2): 707-718.
121. Kwon J J, Nabinger S C, Vega Z, et al. Pathophysiological role of microRNA-29 in pancreatic cancer stroma[J]. Scientific reports, 2015, 5: 11450.
122. Wang K, Sun Y, Tao W, et al. Androgen receptor (AR) promotes clear cell renal cell carcinoma (ccRCC) migration and invasion via altering the circHIAT1/miR-195-5p/29a-3p/29c-3p/CDC42 signals[J]. Cancer letters, 2017, 394: 1-12.
123. Kato M, Kurozumi A, Goto Y, et al. Regulation of metastasis-promoting LOXL2 gene expression by antitumor microRNAs in prostate cancer[J]. Journal of human genetics, 2017, 62(1): 123.
124. Pasqualini L , Bu H , Puhr M , et al. miR-22 and miR-29a are members of the androgen receptor cistrome modulating LAMC1 and Mcl-1 in prostate cancer[J]. Molecular Endocrinology, 2015, 29(7):1037-54.
125. Li R, Liu J, Li Q, et al. miR-29a suppresses growth and metastasis in papillary thyroid carcinoma by targeting AKT3[J]. Tumor Biology, 2016, 37(3): 3987-3996.
126. Saha M N, Abdi J, Yang Y, et al. MiRNA-29a as a tumor suppressor mediates PRIMA-1^Met^-induced anti-myeloma activity by targeting c-Myc[J]. Oncotarget, 2016, 7(6): 7149.
127. Gao S, Cheng C, Chen H, et al. IGF1 3′ UTR functions as a ceRNA in promoting angiogenesis by sponging miR-29 family in osteosarcoma[J]. Journal of molecular histology, 2016, 47(2): 135-143.
128. Qiu F, Sun R, Deng N, et al. miR-29a/b enhances cell migration and invasion in nasopharyngeal carcinoma progression by regulating SPARC and COL3A1 gene expression[J]. PloS one, 2015, 10(3): e0120969.
129. Liu C, Duan P, Li B, Huang C, Jing Y, Yan W. miR-29a activates Hes1 by targeting Nfia in esophageal carcinoma cell line TE-1.Oncol Lett. 2015;9(1):96-102.
130. Zaidi S K, Perez A W, White E S, et al. An AML1-ETO/miR-29b-1 regulatory circuit modulates phenotypic properties of acute myeloid leukemia cells[J]. Oncotarget, 2017, 8(25): 39994.
131. Mundy-Bosse B L, Scoville S D, Chen L, et al. MicroRNA-29b mediates altered innate immune development in acute leukemia[J]. The Journal of clinical investigation, 2016, 126(12): 4404-4416.
132. Kirimura S, Kurata M, Nakagawa Y, et al. Role of microRNA-29b in myelodysplastic syndromes during transformation to overt leukaemia[J]. Pathology, 2016, 48(3): 233-241.
133. Wang H, Ding Q, Wang M, et al. miR-29b inhibits the progression of multiple myeloma through downregulating FOXP1[J]. Hematology, 2019, 24(1): 32-38.
134. Amodio N, Stamato M A, Gullà A M, et al. Therapeutic targeting of miR-29b/HDAC4 epigenetic loop in multiple myeloma[J]. Molecular cancer therapeutics, 2016, 15(6): 1364-1375.
135. Xie J, Wang J, Zhu B. Genistein inhibits the proliferation of human multiple myeloma cells through suppression of nuclear factor-kB and upregulation of microRNA‑29b[J]. Molecular medicine reports, 2016, 13(2): 1627-1632.
136. Jagannathan S, Vad N, Vallabhapurapu S, et al. MiR-29b replacement inhibits proteasomes and disrupts aggresome+ autophagosome formation to enhance the antimyeloma benefit of bortezomib[J]. Leukemia, 2015, 29(3): 727.
137. Zhao X, Liu Y, Li Z, et al. Linc00511 acts as a competing endogenous RNA to regulate VEGFA expression through sponging hsa‐miR‐29b‐3p in pancreatic ductal adenocarcinoma[J]. Journal of cellular and molecular medicine, 2018, 22(1): 655-667.
138. Kohnken R, Wen J, Mundy-Bosse B, et al. Diminished microRNA-29b level is associated with BRD4-mediated activation of oncogenes in cutaneous T-cell lymphoma[J]. Blood, 2018, 131(7): 771-781.
139. Jauhari A, Singh T, Yadav S. Expression of miR-145 and Its Target Proteins Are Regulated by miR-29b in Differentiated Neurons[J]. Molecular neurobiology, 2018, 55(12): 8978-8990.
140. Andrews M C, Cursons J, Hurley D G, et al. Systems analysis identifies miR-29b regulation of invasiveness in melanoma[J]. Molecular cancer, 2016, 15(1): 72.
141. Ding D, Li C, Zhao T, et al. LncRNA H19/miR-29b-3p/PGRN axis promoted epithelial-mesenchymal transition of colorectal cancer cells by acting on Wnt signaling[J]. Molecules and cells, 2018, 41(5): 423.
142. Kim Y J, Hwang K C, Kim S W, et al. Potential miRNA-target interactions for the screening of gastric carcinoma development in gastric adenoma/dysplasia[J]. International journal of medical sciences, 2018, 15(6): 610.
143. Kong Y, Zou S, Yang F, et al. RUNX3-mediated up-regulation of miR-29b suppresses the proliferation and migration of gastric cancer cells by targeting KDM2A[J]. Cancer letters, 2016, 381(1): 138-148.
144. Zhang H, Cheng Y, Jia C, et al. MicroRNA-29s could target AKT2 to inhibit gastric cancer cells invasion ability[J]. Medical Oncology, 2015, 32(1): 342.
145. Cui H, Wang L, Gong P, et al. Deregulation between miR-29b/c and DNMT3A is associated with epigenetic silencing of the CDH1 gene, affecting cell migration and invasion in gastric cancer[J]. PloS one, 2015, 10(4): e0123926.
146. Li C, Dong Q, Che X, et al. MicroRNA-29b-2-5p inhibits cell proliferation by directly targeting Cbl-b in pancreatic ductal adenocarcinoma[J]. BMC cancer, 2018, 18(1): 681.
147. Wang L H, Huang J, Wu C R, et al. Downregulation of miR‑29b targets DNMT3b to suppress cellular apoptosis and enhance proliferation in pancreatic cancer[J]. Molecular medicine reports, 2018, 17(2): 2113-2120.
148. Sun G, Lu J, Zhang C, et al. MiR-29b inhibits the growth of glioma via MYCN dependent way[J]. Oncotarget, 2017, 8(28): 45224.
149. Shi C, Ren L, Sun C, et al. miR-29a/b/c function as invasion suppressors for gliomas by targeting CDC42 and predict the prognosis of patients[J]. British journal of cancer, 2017, 117(7): 1036.
150. Ouyang Q, Chen G, Zhou J, et al. Neurotensin signaling stimulates glioblastoma cell proliferation by upregulating c-Myc and inhibiting miR-29b-1 and miR-129-3p[J]. Neuro-oncology, 2015, 18(2): 216-226.
151. Chung H J, Choi Y E, Kim E S, et al. miR-29b attenuates tumorigenicity and stemness maintenance in human glioblastoma multiforme by directly targeting BCL2L2[J]. Oncotarget, 2015, 6(21): 18429.
152. Liu Y, Zhang J, Sun X, et al. Down-regulation of miR-29b in carcinoma associated fibroblasts promotes cell growth and metastasis of breast cancer[J]. Oncotarget, 2017, 8(24): 39559.
153. Drago-Ferrante R, Pentimalli F, Carlisi D, et al. Suppressive role exerted by microRNA-29b-1-5p in triple negative breast cancer through SPIN1 regulation[J]. Oncotarget, 2017, 8(17): 28939.
154. Li Y, Cai B, Shen L, et al. MiRNA-29b suppresses tumor growth through simultaneously inhibiting angiogenesis and tumorigenesis by targeting Akt3[J]. Cancer letters, 2017, 397: 111-119.
155. Zhao H, Wilkie T, Deol Y, et al. miR-29b defines the pro-/anti-proliferative effects of S100A7 in breast cancer[J]. Molecular cancer, 2015, 14(1): 11.
156. Hou M, Zuo X, Li C, et al. Mir-29b regulates oxidative stress by targeting SIRT1 in ovarian cancer cells[J]. Cellular Physiology and Biochemistry, 2017, 43(5): 1767-1776.
157. Teng Y, Zuo X, Hou M, et al. A Double-negative feedback interaction between MicroRNA-29b and DNMT3A/3B contributes to ovarian cancer progression[J]. Cellular Physiology and Biochemistry, 2016, 39(6): 2341-2352.
158. Teng Y, Zhang Y, Qu K, et al. MicroRNA-29B (mir-29b) regulates the Warburg effect in ovarian cancer by targeting AKT2 and AKT3[J]. Oncotarget, 2015, 6(38): 40799.
159. Lv M, Zhong Z, Huang M, et al. lncRNA H19 regulates epithelial–mesenchymal transition and metastasis of bladder cancer by miR-29b-3p as competing endogenous RNA[J]. Biochimica et Biophysica Acta (BBA)-Molecular Cell Research, 2017, 1864(10): 1887-1899.
160. Taylor M A, Wappett M, Delpuech O, et al. Enhanced MAPK signaling drives ETS1-mediated induction of miR-29b leading to downregulation of TET1 and changes in epigenetic modifications in a subset of lung SCC[J]. Oncogene, 2016, 35(33): 4345.
161. Langsch S, Baumgartner U, Haemmig S, et al. miR-29b mediates NF-κB signaling in KRAS-induced non–small cell lung cancers[J]. Cancer research, 2016, 76(14): 4160-4169.
162. Pastuszak-Lewandoska D, Kordiak J, Czarnecka K H, et al. Expression analysis of three miRNAs, miR-26a, miR-29b and miR-519d, in relation to MMP-2 expression level in non-small cell lung cancer patients: a pilot study[J]. Medical Oncology, 2016, 33(8): 96.
163. Wu D W, Hsu N Y, Wang Y C, et al. c-Myc suppresses microRNA-29b to promote tumor aggressiveness and poor outcomes in non-small cell lung cancer by targeting FHIT[J]. Oncogene, 2015, 34(16): 2072.
164. Li Y, Zhang Z, Xiao Z, et al. Chemotherapy-mediated miR-29b expression inhibits the invasion and angiogenesis of cervical cancer[J]. Oncotarget, 2017, 8(9): 14655.
165. Kollinerová S, Dostál Z, Modrianský M. MicroRNA hsa-miR-29b potentiates etoposide toxicity in HeLa cells via down-regulation of Mcl-1[J]. Toxicology in Vitro, 2017, 40: 289-296.
166. Chen H X, Xu X X, Zhang Z, et al. MicroRNA-29b inhibits angiogenesis by targeting VEGFA through the MAPK/ERK and PI3K/Akt signaling pathways in endometrial carcinoma[J]. Cellular Physiology and Biochemistry, 2017, 41(3): 933-946.
167. Habata S, Iwasaki M, Sugio A, et al. BAG3 increases the invasiveness of uterine corpus carcinoma cells by suppressing miR‑29b and enhancing MMP2 expression[J]. Oncology reports, 2015, 33(5): 2613-2621.
168. Kato M, Kurozumi A, Goto Y, et al. Regulation of metastasis-promoting LOXL2 gene expression by antitumor microRNAs in prostate cancer[J]. Journal of human genetics, 2017, 62(1): 123.
169. Takayama K, Misawa A, Suzuki T, et al. TET2 repression by androgen hormone regulates global hydroxymethylation status and prostate cancer progression[J]. Nature communications, 2015, 6: 8219.
170. Zhu K, Liu L, Zhang J, et al. MiR-29b suppresses the proliferation and migration of osteosarcoma cells by targeting CDK6[J]. Protein & cell, 2016, 7(6): 434-444.
171. Gigante M, Pontrelli P, Herr W, et al. miR-29b and miR-198 overexpression in CD8+ T cells of renal cell carcinoma patients down-modulates JAK3 and MCL-1 leading to immune dysfunction[J]. Journal of translational medicine, 2016, 14(1): 84.
172. Xu Y, Zhu J, Lei Z, et al. Expression and functional role of miR-29b in renal cell carcinoma[J]. International journal of clinical and experimental pathology, 2015, 8(11): 14161.
173. Qiu F, Sun R, Deng N, et al. miR-29a/b enhances cell migration and invasion in nasopharyngeal carcinoma progression by regulating SPARC and COL3A1 gene expression[J]. PloS one, 2015, 10(3): e0120969.
174. García-Vázquez R, Marchat L A, Ruíz-García E, et al. MicroRNA-143 is Associated With Pathological Complete Response and Regulates Multiple Signaling Proteins in Breast Cancer[J]. Technology in cancer research & treatment, 2019, 18: 1533033819827309.
175. Chen J, Chen X. MYBL2 is targeted by miR-143-3p and regulates breast cancer cell proliferation and apoptosis[J]. Oncology Research Featuring Preclinical and Clinical Cancer Therapeutics, 2018, 26(6): 913-922.
176. Xia C , Yang Y , Kong F , et al. MiR-143-3p inhibits the proliferation, cell migration and invasion of human breast cancer cells by modulating the expression of MAPK7[J]. Biochimie, 2018, 147:98-104.
177. Tavanafar F, Safaralizadeh R, Hosseinpour-Feizi M A, et al. Restoration of miR-143 expression could inhibit migration and growth of MDA-MB-468 cells through down-regulating the expression of invasion-related factors[J]. Biomedicine & Pharmacotherapy, 2017, 91: 920-924.
178. Zhou L L , Dong J L , Huang G , et al. MicroRNA-143 inhibits cell growth by targeting ERK5 and MAP3K7 in breast cancer[J]. Brazilian Journal of Medical and Biological Research, 2017, 50(8).
179. Yang Z, Chen D, Nie J, et al. MicroRNA‑143 targets CD44 to inhibit breast cancer progression and stem cell-like properties[J]. Molecular medicine reports, 2016, 13(6): 5193-5199.
180. Zhang F, Cao H. MicroRNA‑143‑3p suppresses cell growth and invasion in laryngeal squamous cell carcinoma via targeting the k‑Ras/Raf/MEK/ERK signaling pathway[J]. International journal of oncology, 2019, 54(2): 689-701.
181. Xu B, Liu J, Xiang X, et al. Expression of miRNA-143 in Pancreatic Cancer and Its Clinical Significance[J]. Cancer biotherapy & radiopharmaceuticals, 2018, 33(9): 373-379.
182. Guo L, Fu J, Sun S, et al. MicroRNA-143-3p inhibits colorectal cancer metastases by targeting ITGA6 and ASAP3[J]. Cancer science, 2019, 110(2): 805-816.
183. Huang F, Wen C, Zhuansun Y, et al. A novel long noncoding RNA OECC promotes colorectal cancer development and is negatively regulated by miR-143-3p[J]. Biochemical and biophysical research communications, 2018, 503(4): 2949-2955.
184. Gomes S E, Pereira D M, Roma-Rodrigues C, et al. Convergence of miR-143 overexpression, oxidative stress and cell death in HCT116 human colon cancer cells[J]. PloS one, 2018, 13(1): e0191607.
185. Gomes S E, Pereira D M, Roma-Rodrigues C, et al. Convergence of miR-143 overexpression, oxidative stress and cell death in HCT116 human colon cancer cells[J]. PloS one, 2018, 13(1): e0191607.
186. Hu Y, Ma Z, He Y, et al. PART-1 functions as a competitive endogenous RNA for promoting tumor progression by sponging miR-143 in colorectal cancer[J]. Biochemical and biophysical research communications, 2017, 490(2): 317-323.
187. Wang G, Gu J, Gao Y. MicroRNA target for MACC1 and CYR61 to inhibit tumor growth in mice with colorectal cancer[J]. Tumor Biology, 2016, 37(10): 13983-13993.
188. Yu B, Liu X, Chang H. MicroRNA-143 inhibits colorectal cancer cell proliferation by targeting MMP7[J]. Minerva medica, 2017, 108(1): 13-19.
189. Gomes S E, Simões A E S, Pereira D M, et al. miR-143 or miR-145 overexpression increases cetuximab-mediated antibody-dependent cellular cytotoxicity in human colon cancer cells[J]. Oncotarget, 2016, 7(8): 9368.
190. Pekow J.et al. Tumor suppressors miR-143 and miR-145 and predicted target proteins API5, ERK5, K-RAS, and IRS-1 are differentially expressed in proximal and distal colon, Am J Physiol Gastrointest Liver Physiol,2015; 308(3): G179-87.
191. Cui J, Wei C, Deng L, et al. MicroRNA‑143 increases cell apoptosis in myelodysplastic syndrome through the Fas/FasL pathway both in vitro and in vivo[J]. International journal of oncology, 2018, 53(5): 2191-2199.
192. Lam J, van den Bosch M, Wegrzyn J, et al. miR-143/145 differentially regulate hematopoietic stem and progenitor activity through suppression of canonical TGFβ signaling[J]. Nature communications, 2018, 9(1): 2418.
193. Liang B, Song Y, Zheng W, et al. miRNA143 Induces K562 Cell Apoptosis Through Downregulating BCR-ABL[J]. Medical science monitor: international medical journal of experimental and clinical research, 2016, 22: 2761.
194. Sun X, Dai G, Yu L, et al. miR-143-3p inhibits the proliferation, migration and invasion in osteosarcoma by targeting FOSL2[J]. Scientific reports, 2018, 8(1): 606.
195. Dong X, Lv B, Li Y, et al. MiR-143 regulates the proliferation and migration of osteosarcoma cells through targeting MAPK7[J]. Archives of biochemistry and biophysics, 2017, 630: 47-53.
196. DiStefano J K. Angiopoietin-like 8 (ANGPTL8) expression is regulated by miR-143-3p in human hepatocytes[J]. Gene, 2019, 681: 1-6.
197. Tang H, Li X, Yang R. Downregulation of microRNA-143 promotes cell proliferation by regulating PKCε in hepatocellular carcinoma cells[J]. Molecular medicine reports, 2017, 16(4): 4348-4354.
198. Chen L, Yao H, Wang K, et al. Long non‐coding RNA MALAT1 regulates ZEB1 expression by sponging miR‐143‐3p and promotes hepatocellular carcinoma progression[J]. Journal of cellular biochemistry, 2017, 118(12): 4836-4843.
199. Li W, Wu H, Li Y, et al. MicroRNA-143 promotes apoptosis of osteosarcoma cells by caspase-3 activation via targeting Bcl-2[J]. Biomedicine & pharmacotherapy, 2016, 80: 8-15.
200. Liu X, Gong J, Xu B. miR-143 down-regulates TLR2 expression in hepatoma cells and inhibits hepatoma cell proliferation and invasion[J]. International journal of clinical and experimental pathology, 2015, 8(10): 12738.
201. Guoping M , Ran L , Yanru Q . miR-143 Inhibits Cell Proliferation of Gastric Cancer Cells Through Targeting GATA6[J]. Oncology Research Featuring Preclinical and Clinical Cancer Therapeutics, 2018, 26(7):1023-1029.
202. Lei C, Du F, Sun L, et al. miR-143 and miR-145 inhibit gastric cancer cell migration and metastasis by suppressing MYO6[J]. Cell death & disease, 2017, 8(10): e3101.
203. Wang F, Liu J, Zou Y, et al. MicroRNA-143-3p, up-regulated in H. pylori-positive gastric cancer, suppresses tumor growth, migration and invasion by directly targeting AKT2[J]. Oncotarget, 2017, 8(17): 28711.
204. Zhang Q, Feng Y, Liu P, et al. MiR-143 inhibits cell proliferation and invasion by targeting DNMT3A in gastric cancer[J]. Tumor Biology, 2017, 39(7): 1010428317711312.
205. An Q, Zhou L, Xu N. Long noncoding RNA FOXD2-AS1 accelerates the gemcitabine-resistance of bladder cancer by sponging miR-143[J]. Biomedicine & Pharmacotherapy, 2018, 103: 415-420.
206. Zhang Q, Zhao W, Ye C, et al. Honokiol inhibits bladder tumor growth by suppressing EZH2/miR-143 axis[J]. Oncotarget, 2015, 6(35): 37335.
207. Zhang L, Zhou D, Guan W, et al. Pyridoxine 5′-phosphate oxidase is a novel therapeutic target and regulated by the TGF-β signalling pathway in epithelial ovarian cancer[J]. Cell death & disease, 2017, 8(12): 3214.
208. Zhang H, Li W. Dysregulation of micro-143-3p and BALBP1 contributes to the pathogenesis of the development of ovarian carcinoma[J]. Oncology reports, 2016, 36(6): 3605-3610.
209. Jin X, Chen X, Hu Y, et al. LncRNA‐TCONS_00026907 is involved in the progression and prognosis of cervical cancer through inhibiting miR‐143‐5p[J]. Cancer medicine, 2017, 6(6): 1409-1423.
210. GóMEz-GóMEz Y, Organista-Nava J, OCAdIz-dELGAdO R, et al. The expression of miR-21 and miR-143 is deregulated by the HPV16 E7 oncoprotein and 17β-estradiol[J]. International journal of oncology, 2016, 49(2): 549-558.
211. Sun X , Zhang L . MicroRNA-143 suppresses oral squamous cell carcinoma (OSCC) cell growth, invasion and glucose metabolism through targeting Hexokinase2[J]. Bioscience Reports, 2017:BSR20160404.
212. He M , Zhan M , Chen W , et al. MiR-143-5p Deficiency Triggers EMT and Metastasis by Targeting HIF-1α in Gallbladder Cancer[J]. Cellular Physiology and Biochemistry, 2017:2078-2092.
213. Chu H , Zhong D , Tang J , et al. A functional variant in miR-143 promoter contributes to prostate cancer risk[J]. Archives of Toxicology, 2016, 90(2):403-414.
214. Ma Z, Luo Y, Qiu M. miR-143 Induces the apoptosis of prostate cancer LNCap cells by suppressing Bcl-2 expression[J]. Medical Science Monitor: international medical journal of experimental and clinical research, 2017, 23: 359.
215. Huang F T, Peng J F, Cheng W J, et al. miR-143 targeting TAK1 attenuates pancreatic ductal adenocarcinoma progression via MAPK and NF-κB pathway in vitro[J]. Digestive diseases and sciences, 2017, 62(4): 944-957.
216. Quaranta M T, Spinello I, Paolillo R, et al. Identification of β-dystrobrevin as a direct target of miR-143: involvement in early stages of neural differentiation[J]. PloS one, 2016, 11(5): e0156325.
217. He Z, Yi J, Liu X, et al. MiR-143-3p functions as a tumor suppressor by regulating cell proliferation, invasion and epithelial–mesenchymal transition by targeting QKI-5 in esophageal squamous cell carcinoma[J]. Molecular cancer, 2016, 15(1): 51.
218. Mao Y, Liu J, Zhang D, et al. miR-143 inhibits tumor progression by targeting FAM83F in esophageal squamous cell carcinoma[J]. Tumor Biology, 2016, 37(7): 9009-9022.
219. Fu T G, Wang L, Li W, et al. miR-143 inhibits oncogenic traits by degrading NUAK2 in glioblastoma[J]. International journal of molecular medicine, 2016, 37(6): 1627-1635.
220. Liu J, Qu C B, Xue Y X, et al. MiR-143 enhances the antitumor activity of shikonin by targeting BAG3 expression in human glioblastoma stem cells[J]. Biochemical and biophysical research communications, 2015, 468(1-2): 105-112.
221. Ding Q, Wang Y, Zuo Z, et al. Decreased expression of microRNA-26b in locally advanced and inflammatory breast cancer[J]. Human pathology, 2018, 77: 121-129.
222. Wilke C M, Hess J, Klymenko S V, et al. Expression of mi RNA‐26b‐5p and its target TRPS 1 is associated with radiation exposure in post‐C hernobyl breast cancer[J]. International journal of cancer, 2018, 142(3): 573-583.
223. Ma X, Dong W, Su Z, et al. Functional roles of sialylation in breast cancer progression through miR-26a/26b targeting ST8SIA4[J]. Cell death & disease, 2016, 7(12): e2561.
224. Xia M, Duan M L, Tong J H, et al. MiR-26b suppresses tumor cell proliferation, migration and invasion by directly targeting COX-2 in lung cancer[J]. Eur Rev Med Pharmacol Sci, 2015, 19(24): 4728-4737.
225. Chen C Y A , Chang J T , Ho Y F , et al. MiR-26 down-regulates TNF-α/NF-κB signalling and IL-6 expression by silencing HMGA1 and MALT1[J]. Nucleic Acids Research, 2016, 44(8):3772-3787.
226. Liang N, Zhou X, Zhao M, et al. Down-regulation of microRNA-26b modulates non-small cell lung cancer cells chemoresistance and migration through the association of PTEN[J]. Acta Biochim Biophys Sin, 2015, 47(7): 530-538.
227. Chen Z G, Zheng C Y, Cai W Q, et al. MiR-26b mimic inhibits glioma proliferation in vitro and in vivo suppressing COX-2 expression[J]. Oncology Research Featuring Preclinical and Clinical Cancer Therapeutics, 2019, 27(2): 147-155.
228. Wang L, Su J, Zhao Z, et al. MiR-26b reverses temozolomide resistance via targeting Wee1 in glioma cells[J]. Cell Cycle, 2017, 16(20): 1954-1964.
229. Li Y P, Dai W M, Huang Q, et al. Effects of microRNA-26b on proliferation and invasion of glioma cells and related mechanisms[J]. Molecular medicine reports, 2017, 16(4): 4165-4170.
230. Li Y, Sun Z, Liu B, et al. Tumor-suppressive miR-26a and miR-26b inhibit cell aggressiveness by regulating FUT4 in colorectal cancer[J]. Cell death & disease, 2017, 8(6): e2892.
231. Fan D, Lin X, Zhang F, et al. Micro RNA 26b promotes colorectal cancer metastasis by downregulating phosphatase and tensin homolog and wingless‐type MMTV integration site family member 5A[J]. Cancer science, 2018, 109(2): 354-362.
232. Benderska N, Dittrich A L, Knaup S, et al. miRNA-26b overexpression in ulcerative colitis-associated carcinogenesis[J]. Inflammatory bowel diseases, 2015, 21(9): 2039-2051.
233. Wu K, Mu X Y, Jiang J T, et al. miRNA‑26a‑5p and miR‑26b‑5p inhibit the proliferation of bladder cancer cells by regulating PDCD10[J]. Oncology reports, 2018, 40(6): 3523-3532.
234. Miyamoto K, Seki N, Matsushita R, et al. Tumour-suppressive miRNA-26a-5p and miR-26b-5p inhibit cell aggressiveness by regulating PLOD2 in bladder cancer[J]. British journal of cancer, 2016, 115(3): 354.
235. Jia C M, Tian Y Y, Quan L N, et al. miR-26b-5p suppresses proliferation and promotes apoptosis in multiple myeloma cells by targeting JAG1[J]. Pathology-Research and Practice, 2018, 214(9): 1388-1394.
236. Wang Y, Sun B, Zhao X, et al. Twist1-related miR-26b-5p suppresses epithelial-mesenchymal transition, migration and invasion by targeting SMAD1 in hepatocellular carcinoma[J]. Oncotarget, 2016, 7(17): 24383.
237. Feng Y, Zu L L, Zhang L. MicroRNA-26b inhibits the tumor growth of human liver cancer through the PI3K/Akt and NF-κB/MMP-9/VEGF pathways[J]. Oncology reports, 2018, 39(5): 2288-2296.
238. Wang Y , Sun B , Sun H , et al. Regulation of proliferation, angiogenesis and apoptosis in hepatocellular carcinoma by miR-26b-5p[J]. Tumor Biology, 2016, 37(8):10965-10979.
239. Jin Q, Li X J, Cao P G. MicroRNA-26b enhances the radiosensitivity of hepatocellular carcinoma cells by targeting EphA2[J]. The Tohoku journal of experimental medicine, 2016, 238(2): 143-151.
240. Chunlin J , Jianting L , Baoxian L , et al. Mcl-1 Is a Novel Target of miR-26b That Is Associated with the Apoptosis Induced by TRAIL in HCC Cells[J]. BioMed Research International, 2015, 2015:1-9.
241. Li, Meng, Long, et al. MiR-26b inhibits melanoma cell proliferation and enhances apoptosis by suppressing TRAF5-mediated MAPK activation[J]. Biochemical & Biophysical Research Communications, 2016, 471(3):361-367.
242. Tsai M M , Huang H W , Wang C S , et al. MicroRNA-26b inhibits tumor metastasis by targeting the KPNA2/c-jun pathway in human gastric cancer[J]. Oncotarget, 2016, 7(26):39511-39526.
243. Si Y , Zhang H , Ning T , et al. miR-26a/b Inhibit Tumor Growth and Angiogenesis by Targeting the HGF-VEGF Axis in Gastric Carcinoma[J]. Cellular Physiology and Biochemistry, 2017:1670-1683.
244. Li J, Liang Y, Lv H, et al. miR-26a and miR-26b inhibit esophageal squamous cancer cell proliferation through suppression of c-MYC pathway[J]. Gene, 2017, 625: 1-9.
245. Yuan T, Yang Y, Chen J, et al. Regulation of PI3K signaling in T-cell acute lymphoblastic leukemia: a novel PTEN/Ikaros/miR-26b mechanism reveals a critical targetable role for PIK3CD[J]. Leukemia, 2017, 31(11): 2355.
246. Kato M, Kurozumi A, Goto Y, et al. Regulation of metastasis-promoting LOXL2 gene expression by antitumor microRNAs in prostate cancer[J]. Journal of human genetics, 2017, 62(1): 123.
247. Clotaire D Z J, Zhang B, Wei N, et al. MiR-26b inhibits autophagy by targeting ULK2 in prostate cancer cells[J]. Biochemical and biophysical research communications, 2016, 472(1): 194-200.
248. Kato M, Goto Y, Matsushita R, et al. MicroRNA-26a/b directly regulate La-related protein 1 and inhibit cancer cell invasion in prostate cancer[J]. International journal of oncology, 2015, 47(2): 710-718.
249. Kurozumi A, Kato M, Goto Y, et al. Regulation of the collagen cross-linking enzymes LOXL2 and PLOD2 by tumor-suppressive microRNA-26a/b in renal cell carcinoma[J]. International journal of oncology, 2016, 48(5): 1837-1846.
250. Zheng W D, Zhou F L, Lin N. MicroRNA-26b inhibits osteosarcoma cell migration and invasion by down-regulating PFKFB3 expression[J]. Genet Mol Res, 2015, 14(4): 16872-16879.
251. Duan G, Ren C, Zhang Y, et al. MicroRNA-26b inhibits metastasis of osteosarcoma via targeting CTGF and Smad1[J]. Tumor Biology, 2015, 36(8): 6201-6209.
252. Cao J, Guo T, Dong Q, et al. miR-26b is downregulated in human tongue squamous cell carcinoma and regulates cell proliferation and metastasis through a COX-2-dependent mechanism[J]. Oncology reports, 2015, 33(2): 974-980.
253. Fukumoto I, Hanazawa T, Kinoshita T, Kikkawa N, Koshizuka K, Goto Y, Nishikawa R, Chiyomaru T, Enokida H, Nakagawa M, Okamoto Y, Seki N.MicroRNA expression signature of oral squamous cell carcinoma: functional role of microRNA-26a/b in the modulation of novel cancer pathways. Br J Cancer. 2015;112(5):891-900.
